# Marine Natural Products as Models to Circumvent Multidrug Resistance

**DOI:** 10.3390/molecules21070892

**Published:** 2016-07-08

**Authors:** Solida Long, Emília Sousa, Anake Kijjoa, Madalena M. M. Pinto

**Affiliations:** 1 Laboratório de Química Orgânica e Farmacêutica, Departamento de Ciências Químicas, Faculdade de Farmácia, Universidade do Porto, Porto 4050-313, Portugal; up201502099@ff.up.pt (S.L.); madalena@ff.up.pt (M.M.M.P.); 2 Interdisciplinary Centre of Marine and Environmental Research (CIIMAR), Porto 4050-123, Portugal; ankijjoa@icbas.up.pt; 3 Instituto de Ciências Biomédicas Abel Salazar (ICBAS), Universidade do Porto, Porto 4050-123, Portugal

**Keywords:** ABC transporters, P-glycoprotein modulators, marine natural products, multidrug resistance (MDR), synthesis, structure activity relationship (SAR)

## Abstract

Multidrug resistance (MDR) to anticancer drugs is a serious health problem that in many cases leads to cancer treatment failure. The ATP binding cassette (ABC) transporter P-glycoprotein (P-gp), which leads to premature efflux of drugs from cancer cells, is often responsible for MDR. On the other hand, a strategy to search for modulators from natural products to overcome MDR had been in place during the last decades. However, Nature limits the amount of some natural products, which has led to the development of synthetic strategies to increase their availability. This review summarizes the research findings on marine natural products and derivatives, mainly alkaloids, polyoxygenated sterols, polyketides, terpenoids, diketopiperazines, and peptides, with P-gp inhibitory activity highlighting the established structure-activity relationships. The synthetic pathways for the total synthesis of the most promising members and analogs are also presented. It is expected that the data gathered during the last decades concerning their synthesis and MDR-inhibiting activities will help medicinal chemists develop potential drug candidates using marine natural products as models which can deliver new ABC transporter inhibitor scaffolds.

## 1. Introduction

It is well known that cancer figures among the leading causes of morbidity and mortality [1]. Despite many advances in therapy, diagnosis, and prevention, cancer remains a deadly disease. Although the general term cancer covers many different diseases, most disseminated cancers share a common feature of not responding to available chemotherapies. The remorseless onset to resistance to a wide variety of structurally and mechanistically unrelated anticancer drugs, known as multidrug resistance (MDR), is the major obstacle for a successful human cancer therapy [2]. This eventually leads to cancer relapse and death. Some cancers such as gastrointestinal and renal cancers are largely unresponsive to chemotherapy, i.e., they have a high degree of intrinsic MDR, whereas leukemias, lymphomas, ovarian, and breast cancers often respond to initial treatment, but then acquire MDR during the course of the disease [3]. MDR to anticancer drugs is therefore a serious health problem that dramatically affects the efficacy of cancer treatments [4]. MDR is a phenomenon by which cancer cells develop broad resistance to a wide variety of structurally and functionally unrelated compounds which may arise from several mechanisms [5,6] including: (i) decreased cellular drug uptake; (ii) activation of detoxifying enzymes; (iii) alterations in the molecular targets of the drugs; (iv) defective apoptotic pathways; and (v) increased drug efflux [6,7,8,9]. One of the most prominent mechanisms of MDR to cytotoxic drugs is usually associated with the overexpression of ATP-binding cassette (ABC)-transporter proteins in the tumor cells. Due to the increased efflux out of cells, these pumps can lead to a reduced cellular accumulation of anticancer drugs. These proteins mediate MDR not only in drug entry, but also in drug sequestration by lysosomes, phase III metabolism, mechanisms activated after nuclear entry, apoptosis, microenvironment, and signal transduction pathways that lead to the overexpression of genes that codify these ABC transporters [10]. Such proteins belong to a large family of 49 genes, classified in seven subfamilies (A–G), with several transporters involved in MDR [11]. These include P-glycoprotein (P-gp or ABCB1), multidrug resistance associated proteins 1-6 (MRP1-6 or ABCC1-6), and breast cancer resistance protein (BCRP or ABCG2) [12]. P-gp is the best characterized efflux pump mediating MDR due to its broadest substrate specificity and widest tissue and organ distribution. This 170 kDa glycoprotein acts as a drug efflux pump for a wide variety of anticancer agents such as anthracyclines and epidodophyllotoxins, thus limiting their bioavailability and activity. P-gp is encoded by the human *MDR-1* gene, which is located at chromosome 7. It contains 1280 amino acids, arranged in two halves, each encompassing a transmembrane domain (TMD) which spans the membrane and an intracellular nucleotide-binding domain (NBD) [13,14].

Several studies have already correlated P-gp expression with resistance to chemotherapeutic drugs, particularly in leukemia cells [15]. Furthermore, down-regulation of P-gp expression was shown to sensitize several tumor-resistant cell lines to chemotherapeutic drugs. Indeed, the use of antisense or rybozyme targeting *MDR-1* gene has led to the sensitization of acute myeloid leukemia (AML), ovarian, colon, and breast cancer cells to doxorubicin as well as to increase the sensitivity of chronic and AML cells to daunorubicin [16,17].

It was found that P-gp could be expressed in Chinese hamster ovary cells, selected for colchicine resistance, almost 40 years ago, and since then there has been an ongoing effort to develop therapies that could either block or inactivate this transporter to increase the concentration of anticancer drugs within cells [18]. First generation of P-gp inhibitors referred to drugs already in clinical use or under investigation for therapeutic ability e.g., verapamil, quinidine, and cyclosporine A [19]. However, most of the first generation P-gp inhibitors were found to lack selectivity for P-gp and being substrates for other transporters and enzyme systems; this promiscuity resulted in unpredictable pharmacokinetic interactions in the presence of anticancer drugs [20]. Moreover, low affinity for P-gp, associated with the original therapeutic activity, required the use of high doses which resulted in unacceptable toxicity [6,21]. Second generation of P-gp inhibitors were developed, based on the selective optimization of side activity (SOSA) approach, to increase the potency and reduce toxicity, many of which were single enantiomers of the first generation drugs. An example of these is dexverapamil which is an *R*-enantiomer of verapamil. Interestingly, many of the second generation P-gp inhibitors such as valspodar (PSC-833), elacridar (GF 120918), and biricodar, also entered clinical trials [6]. Although some of the second generation inhibitors showed better pharmacological profiles in clinical trials, they still had limited use as P-gp modulators since most of them were found to be substrates of cytochrome P450 3A, thus interfering with the metabolism and excretion of co-administered chemotherapeutic agents [22]. The third generation of P-gp inhibitors, which exhibited P-gp inhibitory activity at nanomolar concentrations, were developed to overcome the referred problems through quantitative structure-activity relationships (QSAR) studies and combinatorial chemistry approaches [23]. Since the third generation of inhibitors that entered clinical trials did not interfere with cytochrome P450 3A4, they did not reveal any interference with pharmacokinetics of anticancer drugs [24]. One of the most popular third generation P-gp inhibitors is tariquidar, however, its phase III studies on non-small cell lung cancer patients were terminated due to its high toxicity [25]. The same unwanted results were observed with other third generation inhibitors like zosuquidar, elacridar, laniquidar, and ontogeny [20,26]. Although phase I and II trials were performed with some third generation P-gp inhibitors, an emerging consensus, in 2010, was that P-gp should be taken off the list of druggable targets due to the failure of zosuquidar to show clinical benefits in phase III clinical trials [27]. Until now, the existing P-gp inhibitors have demonstrated limited clinical success due to their limitations in potency and specificity and also to their interactions with anticancer drugs. Moreover, QSAR and docking studies also could not produce promising hits or leads for safe and effective compounds. As there is a permanent need to identify and validate new antitumor agents, with novel mechanisms of action, more efficient P-gp inhibitors are needed.

Recently, there has been a proposal of a new generation of ABC inhibitors which focuses on natural products and natural product mimics [28,29,30], peptidomimetics [31], surfactants and lipids [32], and dual ligands [20]. While the traditional sources of terrestrial plants and microbes will undoubtedly continue to yield valuable new bioactive agents, it is even more important to explore all available pools of molecular diversity. This strategy to seek out new ecosystems for bioprospecting brings with it the opportunity to discover unprecedented molecular structures, with bioactivities unencumbered by known (and evolving) mechanisms of drug resistance [33]. Marine natural products are one of the most interesting targets for global drug discovery since they not only possess structural and chemical uniqueness but also represent novel scaffolds for drug development [34]. Marine natural products have also been studied and tested alongside with synthetic compounds as ABC transporter inhibitors [29,35,36,37,38]. The development of anticancer drugs from marine compounds is one of the most promising approaches in drug discovery with therapeutic agents in clinical practice, such as the anticancer trabectedin [37]. Recently, marine natural products and their analogs with antitumor activity have been developed, and many of them have shown also ability for ABC modulation. Thus, marine natural products are revealing an interesting potential in this field not only for the possibility of being used in combination with other anticancer drugs but also as dual inhibitors of tumor cell growth and P-gp. Thus, they could be of value in the rational design of analogs with high potential and reduced pharmacokinetic interactions.

There are already several reviews which highlighted the importance of marine natural products in cancer and, in particular, as P-gp modulators [29,35,39,40,41,42,43]. However, this review aims to describe not only the effects of the most valuable scaffolds from marine natural products and analogs in overcoming MDR but also their synthetic pathways. These data along with structure-activity relationship herein described could serve to guide medicinal chemists in pursuing innovative inhibitors of ABC transporters using marine natural products as models.

## 2. Marine Natural Products and Derivatives as Inhibitors of ABC Transporters

### 2.1. Terpenoids

#### 2.1.1. Sipholane Triterpenoids and Derivatives

**Figure 1:**
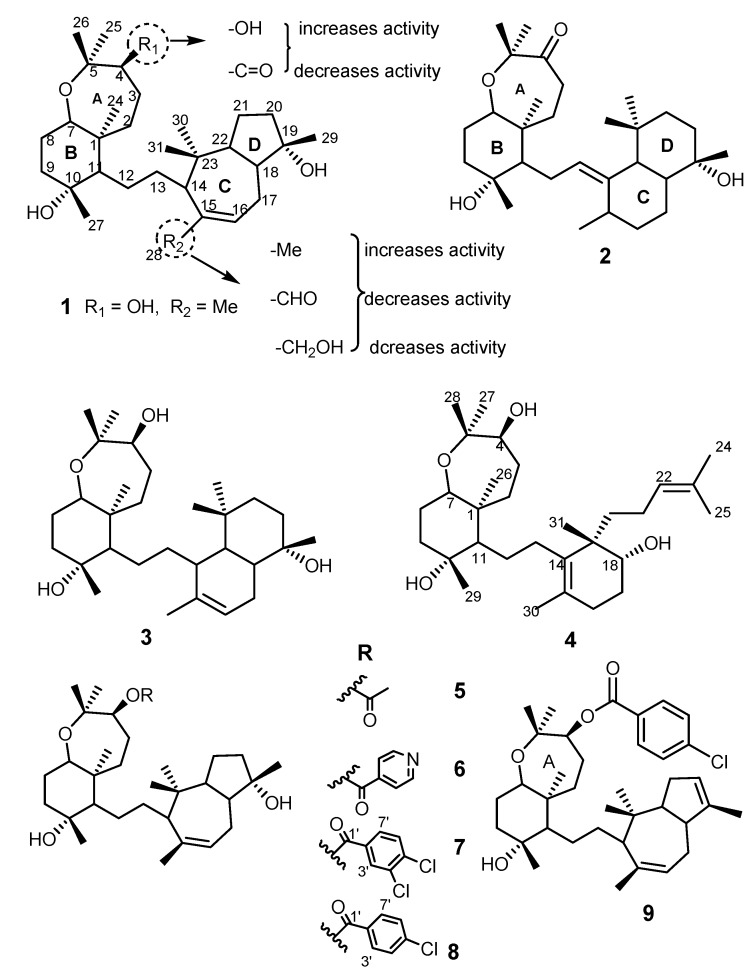
Structures of sipholane triterpenes **1**–**4** and their analogs **5**–**9**. Dashed circles indicate SAR studies performed for MDR activities.

The sipholane triterpenes are compounds that contain a perhydrobenzoxepin (rings A and B) and a [5,3,0] bicyclodecane ring systems (rings C and D) linked together by an ethylene group (Figure 1). This class of triterpenes consists of sipholenol A (**1**), sipholenone E (**2**), sipholenol L (**3**), and siphonellinol D (**4**), isolated from the Red Sea sponge *Callyspongia siphonella* [44,45], and semisynthetic derivatives of sipholenol A such as sipholenol A-4-*O*-acetate (**5**), sipholenol A-4-*O*-isonicotinate (**6**) [39], sipholenol A-4-*O*-3′,4′-dichlorobenzoate (**7**) [46], sipholenol A 4-*O*-4′-chlorobenzoate (**8**), and 19,20-anhydrosipholenol A 4-*O*-4′-chlorobenzoate (**9**) [47] that were synthesized using a ligand-based design approach. Natural and semisynthetic siphonane triterpenoids were shown to reverse MDR activities (**1**–**6**), and display antimigratory and antiproliferative activities in breast cancer cell lines (**7**–**9**). Sipholenol A (**1**) was shown to be potent in reversing MDR KB-C2 [48] and KB-V1 tumor cells with overexpressed P-gp, in a concentration-dependent manner. Compound **1** also inhibited P-gp-mediated drug efflux and did not alter the expression of P-gp after treatment in KB-C2 and KB-V1 cells. This compound stimulated the activity of ATPase, and also inhibited the photo-labeling transporter with [^125^I]-iodoarylazidoprazosin [44]. From SAR studies (Figure 1), it was demonstrated that substitution of the methyl group on C-15 by carbonyl or alcohol reduced the MDR-inhibitory activity, while changing from a hydroxyl to a ketone function at C-4 also reduced the activity [48]. Compounds **2**, **3**, and **4** also enhanced the cytotoxicity of several P-gp substrates including colchicine, vinblastine, and paclitaxel, and reversed the MDR-phenotype in P-gp-overexpressing MDR KB-C2 tumor cells, in a dose-dependent manner [45]. Compound **2** was reported as a better P-gp inhibitor than **1** in KB-3-1 and KB-C2 cell lines [49], while **3** and **4** also showed a reversal of MDR in the same tumor cells, but did not reveal the effect on cells lacking P-gp expression or expressing MRP1, MRP7, or BCRP transporters [36,45,49].

**Scheme 1:**
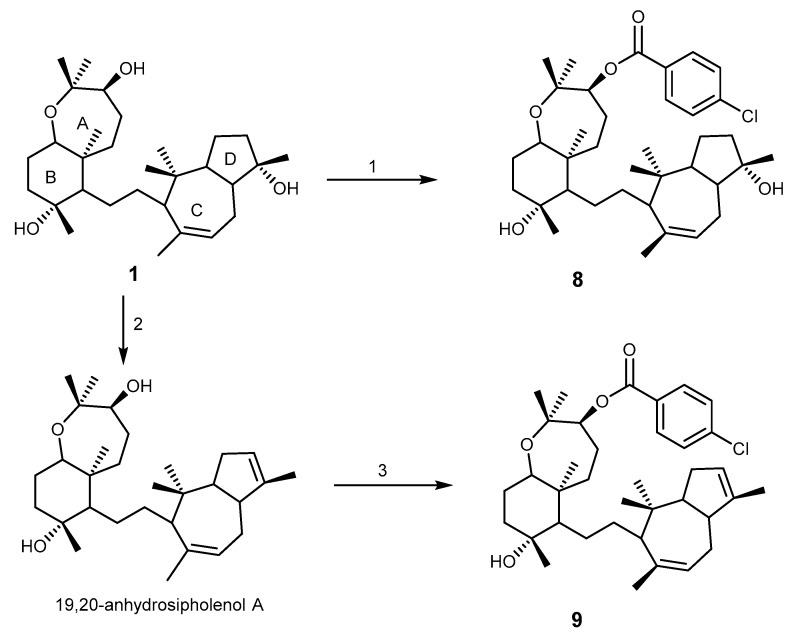
The semi-synthesis transformation of sipholenol A (**1**) into analogs **8** and **9**. *Reagents and conditions*: (1) acid anhydride, MDAP, anhydrous CH_2_Cl_2_; (2) *p*-toluenesulfonic acid, CHCl_3_; (3) acid anhydride, MDAP, anhydrous CH_2_Cl_2_.

The semisynthetic esters **5** and **6** were found to strongly reverse P-gp (ABCB1)-mediated MDR, however they showed no effect on MRP1/ABCC1 and BCRP/ABCG2-mediated MDR. These analogs increased the intracellular accumulation of paclitaxel through inhibition of its active efflux, and re-sensitized cells that have developed drug-resistance to doxorubicin and paclitaxel. These two compounds stimulated more intensively the ATPase activity of P-gp membranes than **2**, **3**, and **4**. In silico molecular docking study, by the inspection of the virtual binding modes of compounds **5** and **6** into human homology model of P-gp, revealed that both compounds were overlapped but with different molecular orientation of ester substituents. Their strong affinity and specificity to P-gp expressing efflux protein indicated that compounds **5** and **6** may represent a potential reversal agents for treatment of MDR cancers [39]. The synthesis of the ester analogs **8** and **9** is presented in Scheme 1, to highlight the strategy involved (using a ligand-based design approach).

#### 2.1.2. Parguerenes and Derivatives

**Figure 2:**
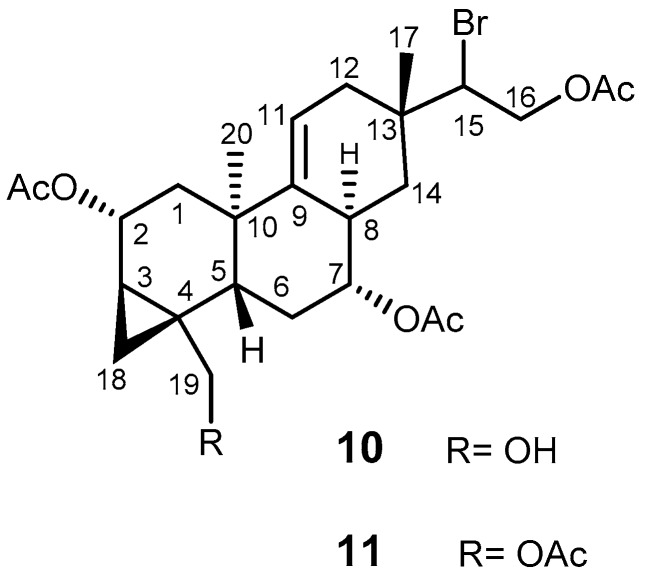
The structures of parguerene I (**10**) and II (**11**).

Parguerenes I (**10**) and II (**11**) (Figure 2) are bromoditerpenes, isolated from the Australian red alga, *Laurencia filiformis* [50]. Several brominated diterpenes of the parguerenes and isoparguerenes isolated from red alga *Jania rubens* were reported to have antitumor, anti-helmintic, and antimicrobial activities [51]. Parguerene derivatives with cytotoxic activity on P388 and HeLa tumor cells possessed an acetoxy group at C-2 and a bromine at C-15 [52]. Compounds **10** and **11** were found to be non-cytotoxic and dose-dependent inhibitors of P-gp mediated drug efflux of verapamil and cyclosporine A. It was also reported that **10** and **11** are capable of reversing P-gp mediated vinblastine, doxorubicin, and paclitaxel in cells overexpressing both P-gp (SW620/ADV300, CEM/VLB100, and HEK93/ABCB1) and MRP1 (2008/MRP1), in a dose-dependent manner. However, their inhibitory effect did not extend to BCRP. Compounds **10** and **11** interact with P-gp by disturbing the extracellular antibody binding epitope of P-gp differently from existing P-gp inhibitors [53]. Therefore, the use of this scaffold as a model for the synthesis of new MDR reversal agents could be of value. To the best of our knowledge, the synthesis of parguerenes has not yet been reported.

### 2.2. Sterols

#### 2.2.1. Agosterol and Derivatives

**Figure 3:**
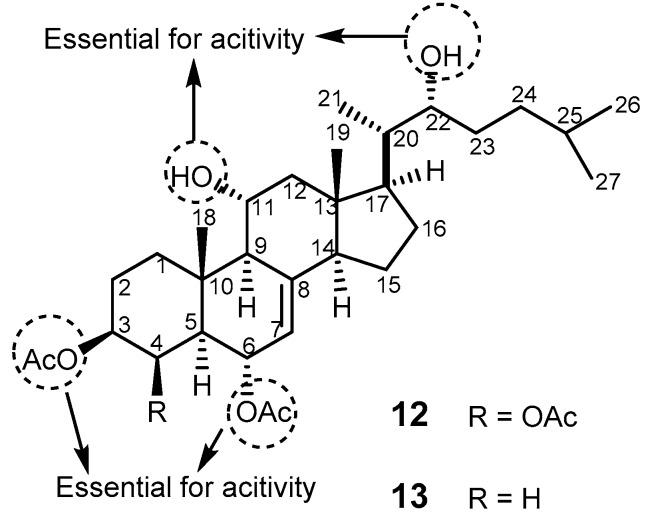
Structure of agosterol A (**12**) and 4-deacetoxyagosterol A (**13**). Dashed circles indicate the groups essential for reversing MDR activity.

Agosterol A (**12**, Figure 3), a polyhydroxylated sterol acetate isolated from the marine sponge *Spongia* sp. [54], was found to completely reverse MDR to colchicine in human carcinoma cells KB-C2 and to vincristine in KB-CV60 (overexpressing MRP1) [55]. Compound **12** was reported to have a dual effect on MRP1 function by reducing MRP1-mediated [^3^H]-LTC4 and enhancing the accumulation of [^3^H]-vincristine in KB/MRP cells to the control levels. It also enhances the ATP-dependent efflux and reduces glutathione intracellular concentration [56]. Therefore, **12** has inhibitory effects on both P-gp and MRP1. The effect of analogs of **12**, including agosterol B, C, A4, D2, A5 and C6, on MDR in tumor cells was also investigated. Agosterol C was found to be a proteasome inhibitor [57]. From the SAR studies, it was possible to infer that the acetoxy groups on C-3, C-4, and C-6, and the hydroxyl groups on C-11 and C-12 were crucial for MDR reversal activity for binding to the C-terminal of MRP1 (Figure 3) [58,59]. 4-Deacetoxyagostrol A (**13**) showed a similar MDR-modulating activity against KB CV-60 cell overexpressing MRP [60].

**Scheme 2:**
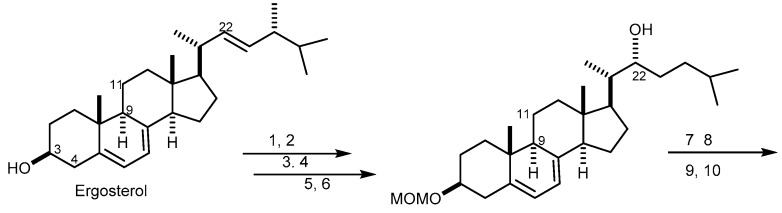

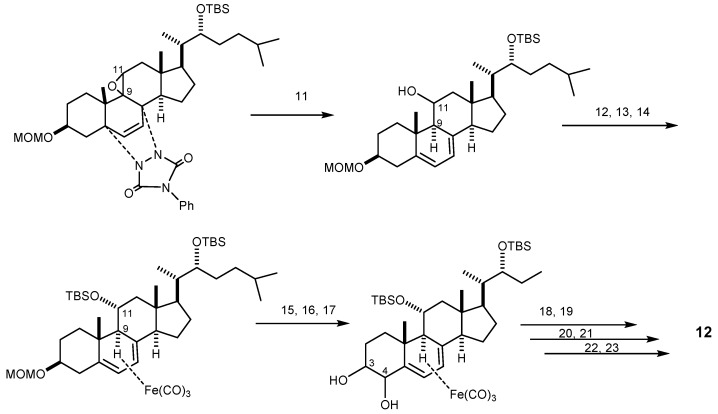
The synthetic pathway of agosterol A (**12**). *Reagents and conditions*: (1) MOMCl, *i*Pr_2_NEt, CH_2_Cl_2_; (2) phthalhydrazide, Pb(OAc)_4_, CH_2_Cl_2_, AcOH, two steps; (3) O_3_,CH_2_Cl_2_, pyridine, then Me_2_S; (4) 3-methylbutylmagnesium bromide, THF, two steps; (5) TMAD, PMe_3_, *p*-OCH_3_BzOH,THF; (6) LiAlH_4_, THF; (7) (*S*)-(−)-MTPA[(*R*)-(+)-MTPA], EDCl.HCl, DMAP, CH_2_Cl_2_; (8)TBSOTf, 2,6-lutidine, DMF-CH_2_Cl_2_; (9) Hg(OAc), EtOH/CHCl_3_/AcOH; (10) 4-phenyl-1,2,4-triazoline-3,5-dione, CH_2_Cl_2_; (11) *m*CPBA, CHCl_3_; (12) LiAlH_4_, THF; (13) TBSOTf, 2,6-lutidine, toluene; (14) Fe(CO)_3_, 1-(4-methyoxyphenyl)-4-pheynyl-1-azabuta-(*E*,*E*)-1,3-diene, PhCH_3_; (15) MgBr_2_-Et_2_O, Me_2_S, CH_2_Cl_2_; (16) TsCl, pyridine, quant.; (17) DBN, PhH, quant.; (18) OsO_4_, pyridine, then aq. NaHSO_3_; (19) TESOTf, pyridine, quant.; (20) Me_3_NO, PhH; (21) BH_3_Me_2_S, THF, then H_2_O_2_, aq. NaOH; (22) TBAF, THF; (23) Ac_2_O, pyridine; HF, pyridine, THF.

The first synthesis of **12** from ergosterol utilizing regioselective epoxy function cleavage and regioselective dehydroxylation as key reactions, and affording a 3.5% yield after 23 steps, was reported in 2001 [61]. The synthetic pathway started with the oxidative cleavage of the C-22/C-23 double bond of ergosterol to introduce a hydroxyl group to C-22, followed by a regioselective reductive epoxy cleavage of the 9α,11α-epoxide to form an 11α-hydroxyl group. The next step was the introduction of a C-3/C-4 double bond which, after a selective dehydroxylation, afforded 3β,4β-dihydroxyl groups. Finally, a differential removal of the protecting group and acetylation afforded **12** (Scheme 2). Compound **13** was also synthesized from ergosterol by reductive regioselective epoxy-cleavage reaction similar to that of **12** [60].

#### 2.2.2. Polyoxygenated Steroids and Derivatives

**Figure 4:**
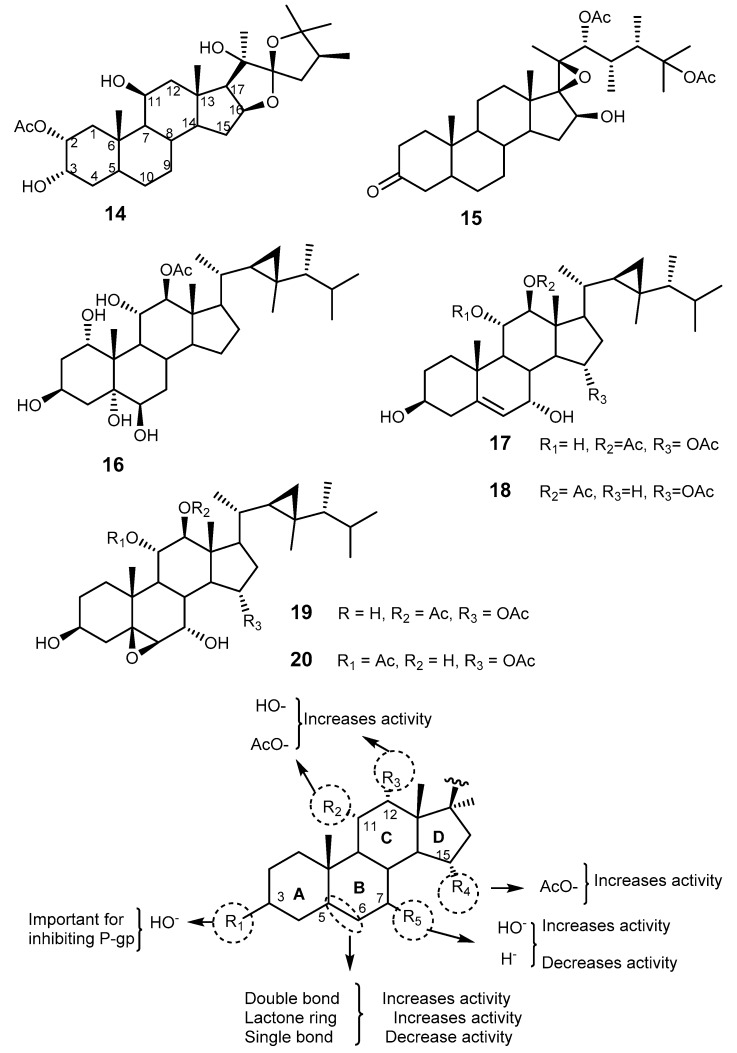
The structures of polyoxygenated steroids **14**–**20**, and SAR for polyoxygenated steroids with antitumor activity in overexpressing P-gp cells.

A number of polyoxygenated steroids has been isolated from gorgonians (*Isis hippuris* [62] and *Leptogorgia sarmentosa* [63]), soft corals (*Sarcophyton* sp. [64] and *Sinularia* sp. [65]), echinoderms, and sponges [62]. Interestingly, all the polyoxygenated steroids investigated for antitumor [66], antibacterial [64], antifungal [64], and reversing MDR [62] activities contain the characteristic 3β,5α,6β-hydroxyl moiety. Among these derivatives are a spiroketal hippurinstanol (**14**), hippuristerone (**15**), which possesses a 3-keto group, and the cyclopropane-containing gorgosterols (**16**–**20**) (Figure 4) [62,64]. Polyoxygenated steroids **14**–**20** also differ in the side chains. Some of them (compounds **16**–**20**) have been tested against KB-C2 overexpressing P-gp cells and showed moderate activity at the concentration of 3 μM by inhibiting the growth of this resistance cell line. Compounds **17** and **18** were the most potent against KB-C2 cells [62].

Semi-synthetic derivatives of 3,16,20-polyoxygenated cholestanes, namely (20*S*)-hydroxy-cholestane-3,6-dione (**21**), (16*S*,20*S*)-dihydroxycholestan-3-one (**22**), (20*S*)-hydroxycholest-1-ene-3,16-dione (**23**), and (20*S*)-hydroxycholest-4-ene-3,16-dione (**24**) were tested for cytotoxicity against three tumor cell lines, i.e., human breast adenocarcinoma (MCF7), human small-cell lung carcinoma (NCI-H187), and human epidermoid carcinoma of cavity (KB).

**Scheme 3:**
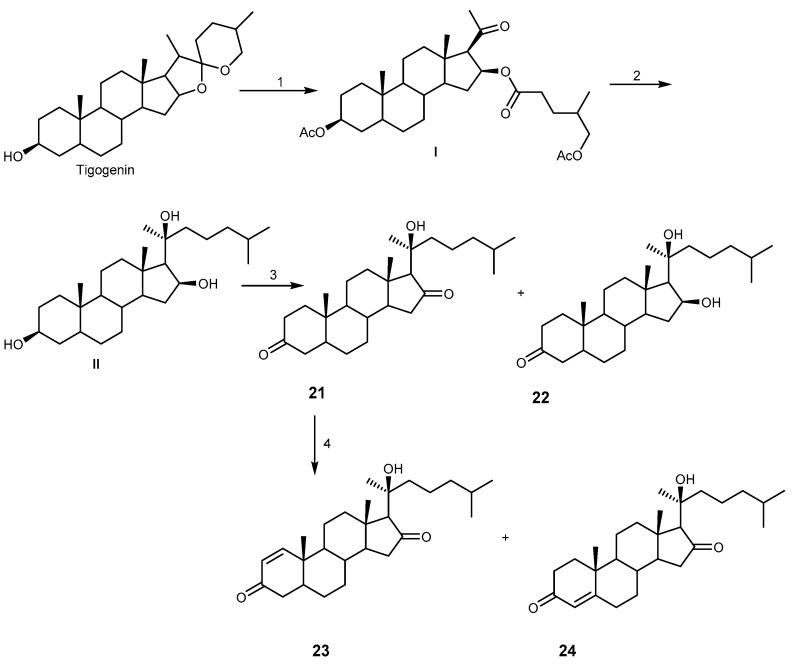
The synthesis of 3,16,20-polyoxygenated steroids **21**–**24**. *Reagents and conditions*: (1) I: NH_4_Cl, pyridine, AcOH; II: CrO_3_, AcOH, H_2_O, (CH_2_Cl_2_)_2_; (2) 4-methylpentylmagnesium bromide, THF; (3) PCC, NaOAc, CH_2_Cl_2_; (4) Ph_2_Se_2_, *m*-iodomybenzoic acid, toluene, reflux.

Compounds **23** and **24** showed strong activity against NCI-H187, and moderate activity against MCF-7 and KB, while compound **21** did not show any activity [63]. Although no MDR-reversal activities were described, these compounds provided a promising synthetic approach for natural polyoxygenated steroids. The synthesis of these four analogs, **21**–**24**, was conducted through four reaction steps, using tigogenin as a starting material, as shown in Scheme 3.

The synthetic pathway started with the transformation of a spiroketal functionality of tigogenin to a keto ester using the method previously described by Micovic et al., and modified by Fushs et al. [67]. The keto ester **I** was then transformed to a trihydroxyl intermediate **II** by Grignard reagent which, after oxidation, gave compounds **21** and **22**. Dehydrogenation of **21** then afforded **23** and **24** [63]. In addition, the introduction of epoxidation and dihydroxylation in suitable positions of steroid nucleus containing oxygenated functions was reported by using transition metal-based oxidants such as methyltrioxorhenium-hydrogen peroxide system, ruthenium tetraoxide, osmium tetraoxide, and potassium permanganate [68].

### 2.3. Polyketides

#### 2.3.1. Bryostatin and Derivatives

Bryostatins are highly oxygenated marine macrolides with a polyacetate backbone [69], and more than 20 analogs have been isolated from the marine bryozoans *Bugula neritina* and *Amathia convuluta* [70]. Most of bryostatins possess antineoplastic activity in many cancer systems like M5076 sarcoma, L10AB cell, and mouse lymphogenous metastatic model SCID. 

**Figure 5:**
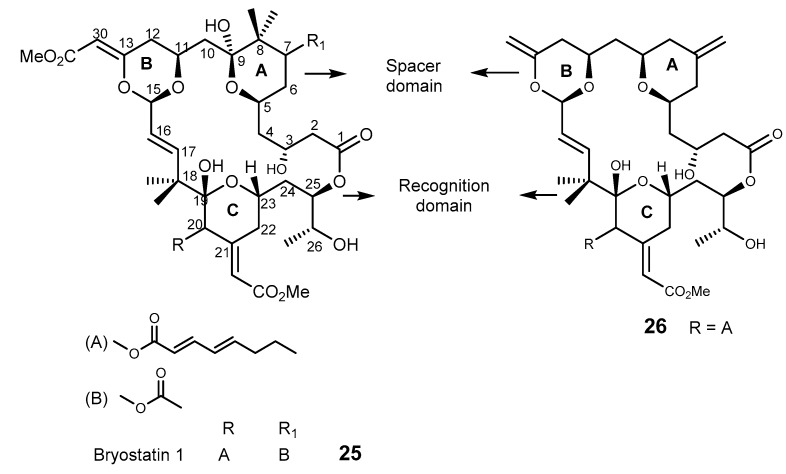
The structures of bryostatin 1 (**25**) and merle 23 (**26**).

Bryostatin 1 (**25**, Figure 5) was reported to down-regulate *MDR-1* and *BCL-2*, but up-regulates *BAX* (induction of apoptosis) [71,72]. Spitaler et al. reported that **25** interacted with the mutated *MDR-1*-V185 and the wild-type *MDR-1*-G185, but reversed MDR and inhibited drug efflux only in P-gp-V185 mutants [72]. The mechanism of regulating MDR for **25** was also reported as protein kinase C (PKC) independent, and it seems to interact directly with *MDR-1* encoded P-gp [73]. However, in a human breast cancer cell line (MCF-7), **25** only decreases P-gp phosphorylation after 24 h treatment in a concentration of 100 nM, but did not affect P-gp function in the intracellular accumulation of [^3^H]-vinblastine and rhodamine 123 [74]. It was found that C-1 to C-9 segment of bryostatins is suitable for both synthetic and biological investigation, and this segment was prepared from an inexpensive and easily accessible compound 2,2-dimethyl-8-oxabicyclo[3,2,1]oct-6-en-3-one [69]. Merle 23 (**26**, Figure 5), is a synthetic analog of bryostatin and whose structure differs from that of bryostatin 1 (**25**) in four positions of the space domain fragment. Interestingly, merle 23 (**26**) showed phorbol ester-like behavior not that of bryostatin in a human prostate cancer cell line (LNCaP), and this finding provided a powerful tool to dissect bryostatins’ mechanisms and responses [75,76].

**Scheme 4:**
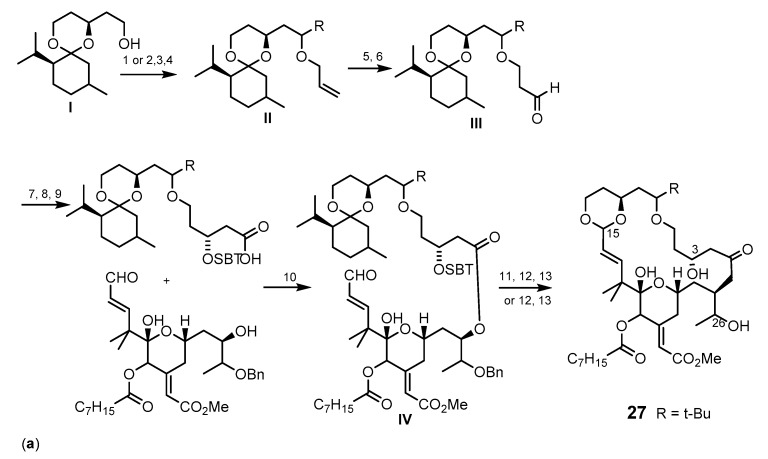

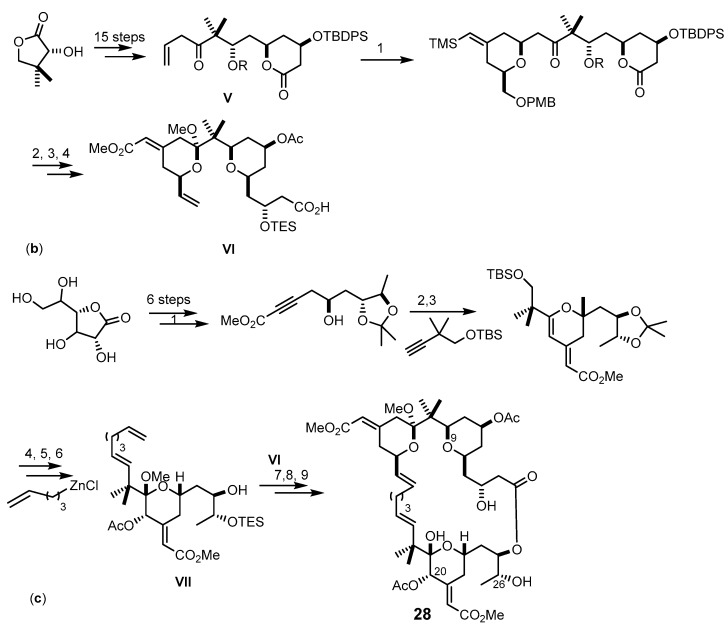
Synthesis of the methyl bryostatin 1 (**25**) analogs **27** (**a**) and **28** (**b**,**c**). *Reagents and conditions*: (**a**) (1) NaH, allybromide, THF, rt; (2) Dess-Martin periodinane, CH_2_Cl_2_, rt; (3) *t*-BuLi, Et_2_O, −78 °C, (recyclable 1:1 diastereomer mixture: Dess-Martin periodinane, CH_2_Cl_2_, rt, then NaBH_4_, CeCl_3·_7H_2_O, −40 °C; (4) *t*-BuOH, allyl bromide, THF, rt; (5) 9-BBN, THF, 66 °C, then NaOH, H_2_O_2_; (6) Dess-Martin periodinane, CH_2_Cl_2_, rt; (7) (−)-(lpc)BOMe, allylmagnesium bromide, CH_2_Cl_2_, −78 °C to rt; (8) TBSCl, imidazole, THF, rt; (9) catalytic KMnO_4_, NaIO_4_, rt; (10) 2,4,6-trichloro-benzoylchloride, Et_3_N, DMAP, CH_2_Cl_2_, rt; (11) HF·pyridine, CH_3_CN, rt; (12) Amberlyst-15 resin, CH_2_Cl_2_, rt; (13) Pd(OH)_2_, H_2_, EtOAc, 1 atm; (**b**) (1) 10 mol % of [CpRu(CH_3_CN)_3_]PF_6_, acetone, rt; (2) NBS, DMF then BF3-OEt_2_, 1,3-propanedithiol, CH_2_Cl_2_, 0 °C then PPTS, CH_3_OH, CH(OCH_3_)_3_, refux; (3) TESCl, DMAP, then pyridine, Ac_2_O; PPTS, MeOH, rt; DMSO, (COCl)_2_, Et_3_N, CH_2_Cl_2_ −78 °C; Ph_3_PCH_3_Br, *n*-BuLi; (4) TBAF, THF, rt; Me_3_SnOH, DCE, 140 °C, microwave; Pd(PPh_3_)_4_, CO, DMF/CH_3_OH, 85 °C; TESOTf, 2,6-lutidine, CH_2_Cl_2_; (**c**) (1) *n*-BuLi, methyl propionate, BF_3_·OEt_2_, THF, −78 °C; (2) Pd(OAc)_2_, tris(2,6-dimethoxyphenyl) phosphine, benzene, then Pd(O_2_CCF_3_)_2_, rt; (3) trifluoroperacetic acid, NaHPO_4_, CH_2_Cl_2_/CH_3_CN/ CH_3_OH, 0 °C; Dess-Martin oxidation; NaBH_4_, CeCl_3_·7H_2_O, −30^o^°C; Ac_2_O, pyridine, DMAP, CH_2_Cl_2_, rt; (4) CrCl_2_, CHI_3_, THF, rt; 26% (47% BRSM); Pd(PPh_3_)_4_, THF, rt; (5) AcOH/H_2_O; (6) TESCl, ETA, DMF, −35 to −15 °C; (7) Et_3_N, DMAP, 2-mehyl-6-nitrobenzoic acid anhydride, CH_2_Cl_2_; (8) benzene, 50–80 °C, 17 mol % of Grubbs-Hoveyda catalyst; (9) PPTS, MeOH, rt.

The synthesis of bryostatin analog **27** (Scheme 4a) without ring A and with simplified ring B was carried out by a convergent esterification-macrotransacetalization procedure to couple the recognition (C-17–C-27) and the spacer (C-1–C-16) domain fragments (Figure 5) [70]. The synthetic pathway involves an allylation of an appropriate alcohol **I** to give the ether intermediate **II**. Hydroboration of the ether intermediate **II**, followed by oxidation gave the appropriate aldehyde **III**, which was then subjected to an oxidative cleavage reaction to furnish the spacer domain. A Yamanguchi esterification was used to produce the next intermediate **IV**, after which macrotranscetalization and deblocking of C-26 alcohol were accomplished to yield bryostatin analog **27** in 11 steps (Scheme 4a) [77].

Trost et al. reported the synthesis of the bryostatin analog **28** with cross-metathesis approach at C-16–C-17 bond using ruthenium and palladium coupling methodologies for the synthesis of B- and C-rings [78,79]. The synthetic pathway of the spacer domain started from (*R*)-pantolactone and a protected alcohol to produce the intermediate **V**. The coupling of the intermediate **V** with an alkyne afforded B-ring pyran. The A-ring ketal was formed by deprotection and cyclization reactions, and the spacer domain **VI** was formed by deprotection, oxidation, and olefination (Scheme 4b). The C-ring fragment was prepared from a lactonized sugar using Roy’s approach [80], and the recognized domain **VII** was obtained as described in Scheme 4c. Finally, a Shiina esterification was used to join **VI** and **VII** to give **28** with 36% yield [79].

#### 2.3.2. Discodermolide

**Figure 6:**
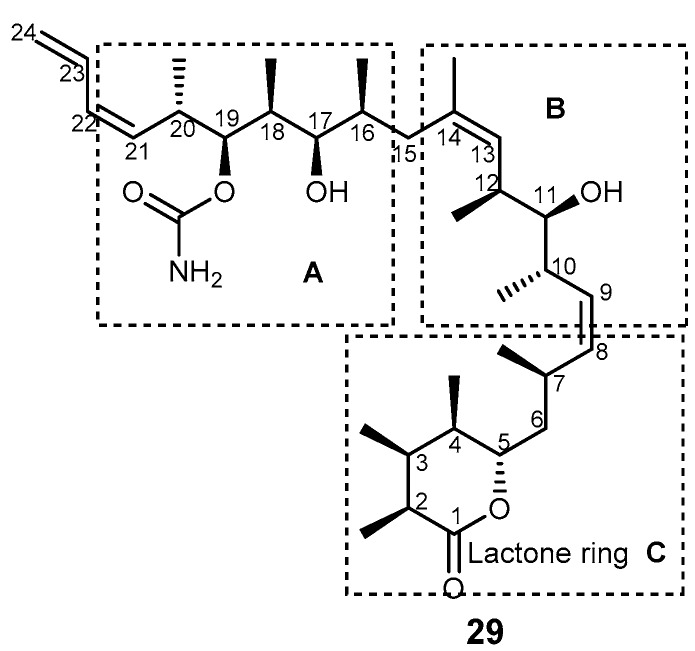
Structure of discodermolide (**29**) with highlighted subunits A, B, and C.

Discodermolide (**29**, Figure 6) is a polyketide found in the marine sponge *Discodermia dissoluta*. This marine product was reported to have a mechanism of action similar to that of taxol, i.e., by blocking the cell cycle at G2/M checkpoint, and inducing apoptosis against several cancer cell lines and against taxol-resistant cells.

Biological activities of **29** include immunosuppressive properties, antiproliferative and antimitotic properties, and was shown to be a potent inducer of accelerated cell senescence, a neuroprotective agent, and to promote tubulin assembly [81,82,83,84]. In addition, **29** was also found to decrease the MDR to taxol in paclitaxel-resistant colon carcinoma (SW60AD-300) and MDR ovarian carcinoma (A2780Ad) [85]. 

**Scheme 5:**
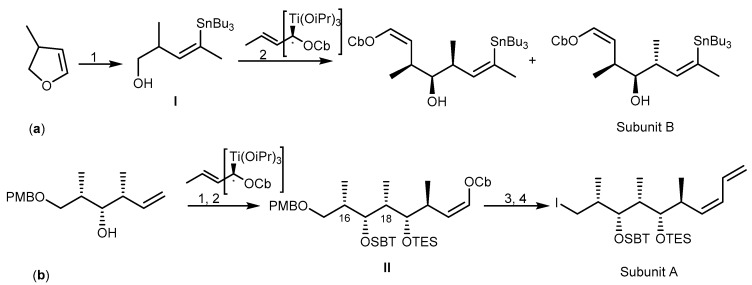

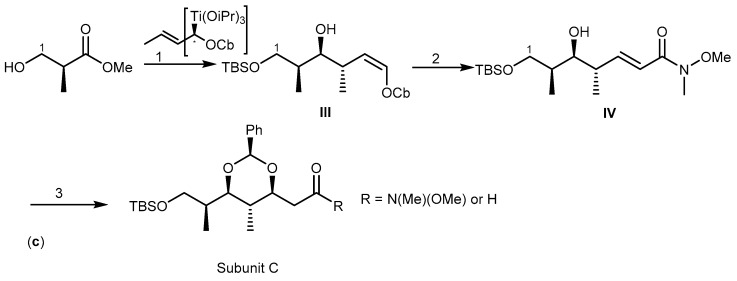
Total synthesis of subunits A, B, and C of (+)-discodermolide (**29**). *Reagents and conditions*: (**a**) (1) *t*-BuLi, Me_2_CuLi, LiCN, Et_2_O/DMS then *n*-Bu_3_SnCl; (2) TEMPO, BAIB; (**b**) (1) TBSOTf, 2,6-lutidine, O_3_, Sudan III then DMS; (2) TBSOTf, 2,6-lutidine; (3) Ni(acac)_2_, (CH_2_=CH_4_)Sn, MeLi; (4) DDQ, CH_2_Cl_2_/H_2_O, I_2_, PPh_3_; (**c**) (1) TBSCl, imidazole, DIBAL-H, CH_2_Cl_2_, (COCl)_2_, DMSO; (2) O_3_, Sudan III, (EtO)_2_P(O)CH_2_C(O)NMe, NaH; (3) PhCHO, KHMDS.

The strategy to synthesize (+)-discodermolide (**29**) and its analogs was achieved by preparing the three subunits: A (C-15–C-24), B (C-8–C-14), and C (C-1–C-7). The synthetic pathway was designed by two key disconnection points at C-7–C-8 and C-14–C-15 with the two *Z* double bonds at C-8–C-9 and C-13–C-14 being pivotal in this strategy. Formation of the C-7–C-8 bond was envisaged through an acetylide addition/reduction sequence, whereas formation of the C-14–C-15 linkage was accomplished by a Pd-catalyzed C(sp^2^)-C(sp^3^) cross-coupling reaction [86]. Subunit B was prepared in two main stages: a crotyl-titanation reaction for the installation of the stereotriad and a dyotropic rearrangement to build a *Z*-double bond **I** (Scheme 5a). The synthesis of subunit A started with the preparation of the C-16–C-18 *syn-syn* stereotriad **II**, planned by means of a substrate-based crotylation reaction under Keck’s conditions (Scheme 5b). The third building block, C-1–C-7 subunit C, incorporates four stereocenters. The approach started with standard Brown crotylation of aldehyde to afford *syn-anti* homoallylic alcohol **III**. Then, the α,β-unsaturated Weinreb amide was obtained through a classical Horner-Wadsworth-Emmons olefination. Finally, reaction of amide **IV** with benzaldehyde yielded subunit C (Scheme 5c). The assembly of the three subunits by cross-coupling reaction and deprotection were the last steps to afford **29**. Recently, (+)-discodermolide (**29**) was synthesized by catalytic stereoselective diene hydroboration, and a strategy for alkylation of chiral enolate reaction was established through 36 steps with 13% yield [87].

#### 2.3.3. Halicondrin B and Derivatives

**Figure 7:**
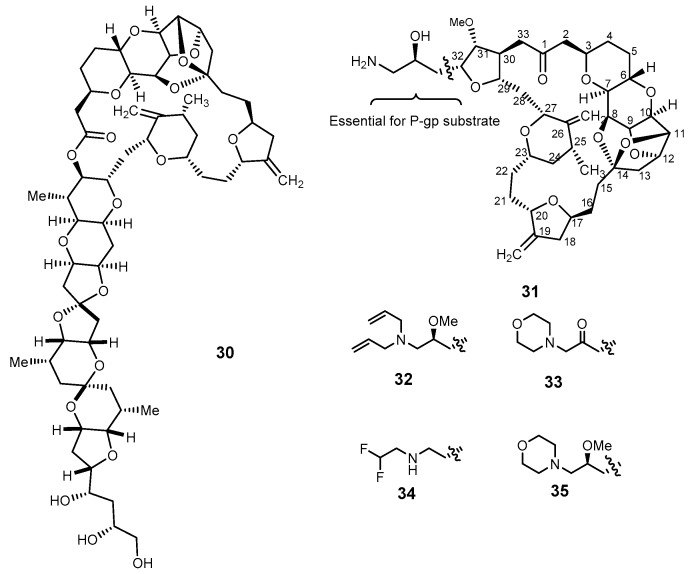
The structure of halichondrin B (**30**), eribulin (**31**), and the analogs **32**–**35**.

The macrolide halichondrin B (**30**, Figure 7), a polyether isolated from the marine sponge *Halichondria okadai*, exhibited both in vitro and in vivo antitumor activity [88]. However, the very limited availability impairs this marine natural product for its therapeutic application [89]. Eribulin mesylate (E7389, or Halaven^®^, **31**), a synthetic analog of **30**, was approved in 2010 by FDA for the treatment of locally advanced and metastatic breast cancer by inhibiting the microtubules [89,90], and was reported as a P-gp substrate [91]. The structure of **31** (Figure 7) is complex, containing several oxygen heterocycles, two exocyclic methylene groups, a ketal group, and a pyran ring with *trans* ring junction, along with 15 stereogenic centers. Several molecular modifications of **31** have been performed, and SAR studies with semi-synthetic analogs revealed that the substituent on C-32 of **31** plays an important role in the P-gp mediated drug efflux. The presence of an amine function in the substituents on C-32 in the analogs **32**–**35** led to a low susceptibility to P-gp-mediated drug efflux, and compounds **32**–**35** were active against MDR tumor cell line in vitro and in xenograft modelsin vivo [43,91]. Furthermore, treatment with eribulin in pretreated metastatic breast cancer patients was confirmed as feasible and safe for real-world patients [92].

**Scheme 6:**
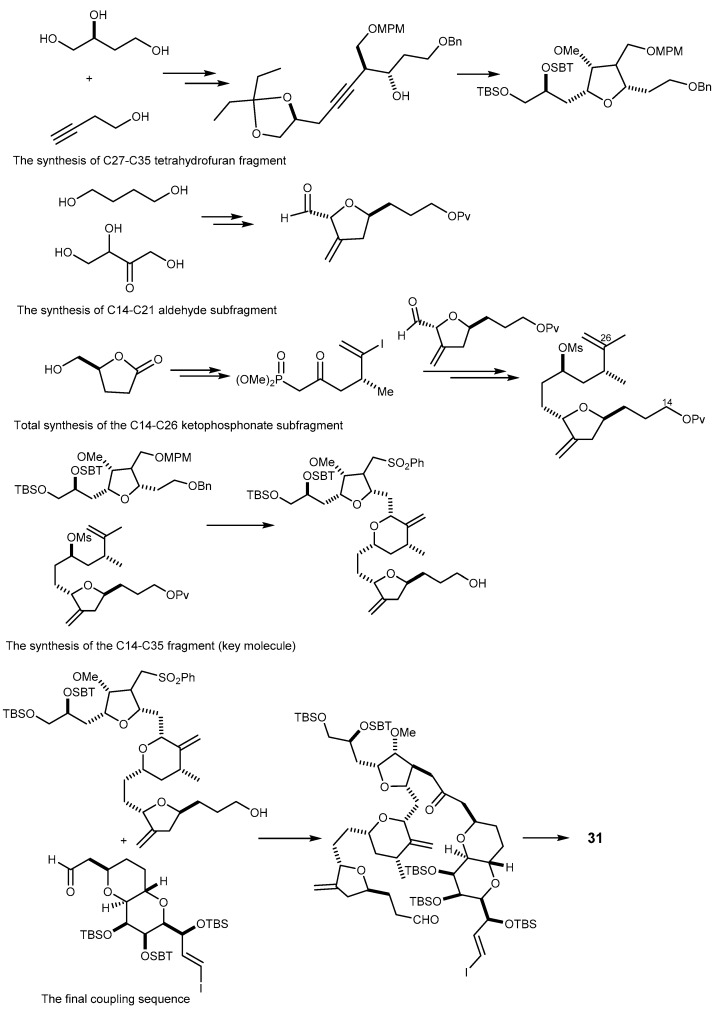
Schematic approach for the total synthesis of eribulin (**31**).

Different methods for the synthesis, to obtain milligram to gram amounts of **31** have been reported [75,93]. Generally, the synthetic pathway of **31** consists of preparation of two fragments, i.e., C-1–C-13 and C-14–C-26, as described by Kishi’s group in 1992, and one key fragment (C-27 to C-35). The final assembly strategy is accomplished by the coupling reaction depicted in Scheme 6 [90,93].

### 2.4. Alkaloids

#### 2.4.1. Lamellarins and Derivatives

**Figure 8:**
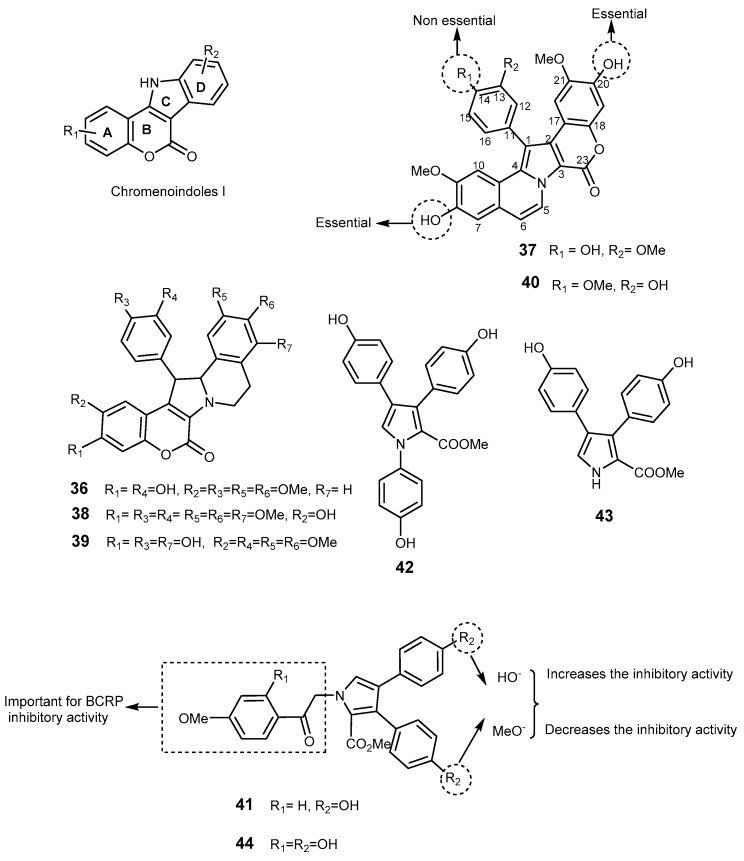
The scaffold of lamellarins and their representatives **36**–**44**. SAR studies for lamellarin D (**37**) and lamellarin O (**41**) regarding antitumor activity on BCRP overexpressing cells.

Lamellarins are a group of polycyclic pyrrole-containing alkaloids possessing a common 14-phenyl-*6H*-[1]-benzopyrano[4′,3′,3,5]pyrrolo-[2,1-α]isoquinoline ring system [94]. Lamellarins were isolated from many marine organisms such as a prosobranch mollusk (*Lamellaria* sp.) [35,95], an ascidian (*Daphniphyllum chartaceum*), a sponge (*Dendrilla cactos*), and unidentified ascidians [35]. More than 50 lamellarins have been described and mainly differ in the number and position of hydroxyl and methoxy groups on the common chromenoindoles I scaffold (Figure 8) [96]. Some lamellarins possess interesting biological activities e.g., lamellarin L (**36**) which exhibits cytotoxicity against P388 and A549 cancer cell lines [97], lamellarin D (**37**) which is also an inhibitor of topoisomerase I-targeted antitumor agents and an antiproliferative agent [96,98] and the immunomodulating lamellarin α-20 sulfate which inhibits also HIV [99]. Lamellarin I (**38**) was the most potent polycyclic pyrrole-containing alkaloid described as a MDR modulator. Methoxy-derivatives of lamellarin D (**37**), lamellarin K (**39**), and lamellarin N (**40**) (Figure 8), exhibit not only potent cytotoxicity against MDR cancer cell lines, but also reverse their MDR at non-cytotoxic concentration [100,101]. Compound **38** was 16 folds more sensitive than verapamil in doxorubicin-resistant Lo Vo/Dx cell line, and it increased the cytotoxicity of doxorubin, vinblastine, and daunorubicine in MDR cells because it directly inhibited P-gp pump function and increased drug accumulation in the cells [102]. Lamellarin O (**41**) is relevant as a multiple P-gp, BCRP, and MRP1 inhibitor [96], but synthetic analogs of **41**, designed by QSAR studies and incorporating the methoxyacetophenone moiety unit, did not show BCRP inhibitory activity [103]. This study also allowed to establish some SAR features: bromo or mono-/dimethyl substituents on the indole moiety (Aryl-R_2_) in lamellarin O (**41**) lead to a decrease in the BCRP inhibitory activity [103] (Figure 8).

The structures of lamellarins are categorized into three types (Figure 8)—type I-a (lamellarin D, **37**), type I-b (lamellarin L, **36**), and type II [lamellarins O (**41**), R (**42**), Q (**43**)]. The syntheses of these derivatives are illustrated by type as presented in the next subsections.

**Scheme 7:**
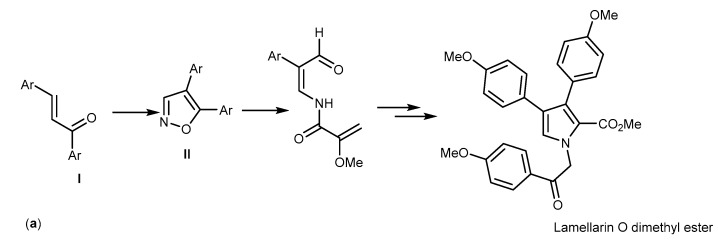

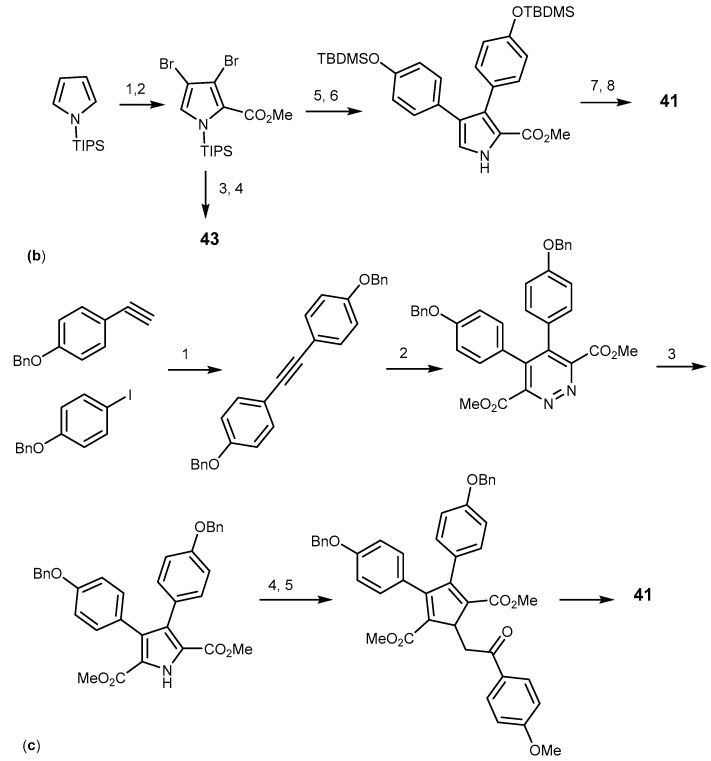
Synthesis of lamellarin O (**41**) and lamellarins type II. *Reagents and conditions*: (**a**) (1) H_2_O_2_, NaOH, EtOH/H_2_O; (2) BF_3_·Et_2_O, Et_2_O, reflux, then NH_2_OH·HCl, reflux, pyridine, EtOH; (3) H_2_, Pd/C, THF; (4) ClCOCO_2_Me, pyridine, THF; (5) Ti-graphide (TiCl:C_8_K = 1:2), DME, reflux; *p*-MeO-C_6_H_4_COCH_2_Br, K_2_CO_3_, acetone, reflux; (**b**) (1) NBS (3 eq), THF; (2) PhLi (1 eq), then ClCO_2_Me (1.05 eq); (3) Pd(PPh_3_)_2_Cl_2_ (10 mol%), 1,4-dioxane, *p*-TBOMSO-C_4_H_6_-SnMe_3_ (2 eq); (4) Bu_4_NF (1.1 eq), THF, then 0.5 M HCl; (5) Bu_4_NF (1.1 eq), THF then 0.5 M HCl; (6) PPd(PPh_3_)_2_Cl_2_ (10 mol %), 1,4-dioxane, *p*-TBOMSO-C_4_H_6_-SnMe_3_ (2 eq); (7) *p*-MeO-C_4_H_6_-COBr (3 eq), K_2_CO_3_ (5 eq), Bu_4_NCl (20 mol %), THF; (8) Bu_4_NF (1.1 eq), THF, then 0.5 M HCl; (**c**) (1) Pd(PP_3_)_2_Cl_2_ (3 mol %), CuI (6 mol %), Et_3_N; (2) dimethyl 1,2,4,5-tetrazine-3,6-dicarboxylate, toluene, reflux; (3) Zn, HOAc; (4) *p*-MeO-C_4_H_6_-COBr, K_2_CO_3_, DMF; (5) LiOH, THF/CH_3_OH/H_2_O (3:2:1); (6) TFA, CH_2_Cl_2_; (7) H_2_,Pd/C (0.1 wt %), EtOH.

A total synthesis of a type-II lamellarins was described by Furstner in 1995. It was the first characterized pathway by a non-fused pyrrole moiety. The synthetic pathways started with the Sheffer-Weitz-epoxidation of an enone **I**, as depicted in Scheme 7a, followed by a Lewis acid-mediated rearrangement and condensation with hydroxylamine to afford the corresponding isoxazole **II**. Cleavage of the N-O bond, condensation, and McMurry cyclization were the subsequent steps to yield lamellarin O dimethyl ester (Scheme 7a) [104]. Another alternative for type-II lamellarin synthesis was performed by cross-coupling using the Stille, Suzuki or Negishi methods, as described by Banwell et al. in 1997. In this process, the pyrrole was protected to form an intermediate which was suitable to afford dibromopyrrole 2-carboxylate. *N*-desilylation and cross-coupling with aryl stannane produced the coupling product which was subjected to *N*-alkylation, followed by desilylation to provide **41** with high yield of 45% in six steps (Scheme 7b) [105]. The Banwell strategy was employed by Alvarez et al. with variations on solid phase chemistry and gave lamellarin **43** (in 13% yield), and lamellarin **41** which could not be isolated [106]. In 2008, Iwao et al. used Banwell’s Suzuki-Miyaura cross-coupling approach to synthesize type II lamellarins, yielding compounds **43**, **41**, and lamellarin P (**44**) in 7, 11, and 12% respectively, over eight steps [107]. Therefore, increasing the steps of reaction did not increase the reaction yield. Boger et al. described the synthesis of lamellarin **41** using an azadiene Diels-Alder reaction named as 1,2,4,5-tetrazine-1,2-diazine-pyrrole transformation with the overall yield of 34% within seven steps [108]. The pyrrole moiety was assembled by a [4+2] cycloaddition/cycloreversion reaction, followed by a reductive ring transformation. They started by forming the acetylenic precursor using Sonogashira-coupling from aryl acetylene and aryl iodide, followed by the cycloaddition of this precursor to give 1,2-diazine which was then subjected to a reductive ring contraction and *N*-alkylation. Selective hydrolysis of the symmetrical diester provided the monoacid. The subsequent hydrogenolysis afforded lamellarin **41** (Scheme 7c) [108]. In 2012, Vazquez et al. used the Paal-Knorr pyrrole synthesis to synthesize **41** in 25% yield in seven steps, and **43** in 28% yield in six steps [94].

The syntheses of lamellarins type I-a were performed via different approaches such as the halogenation and cross-coupling reaction, *N*-ylide-mediated pyrrole ring formation, and other miscellaneous approaches. Lamellarin D (**37**) was synthesized by Alverz et al. [109], starting from the pyrrole ester. The A and B subunits of **37** were established by *N*-alkylation of pyrrole ester by Baeyer-Villiger oxidation, palladium-catalyzed Heck-type cyclization, followed by a regioselective bromination, providing the tricyclic building block which was subjected to Suzuki-Miyaura cross-coupling sequence *O*-isopropylation of the phenolic group and to a second bromination-Suzuki cross-coupling sequence. Introduction of a 5,6-double bond was accomplished by oxidation.

**Scheme 8:**
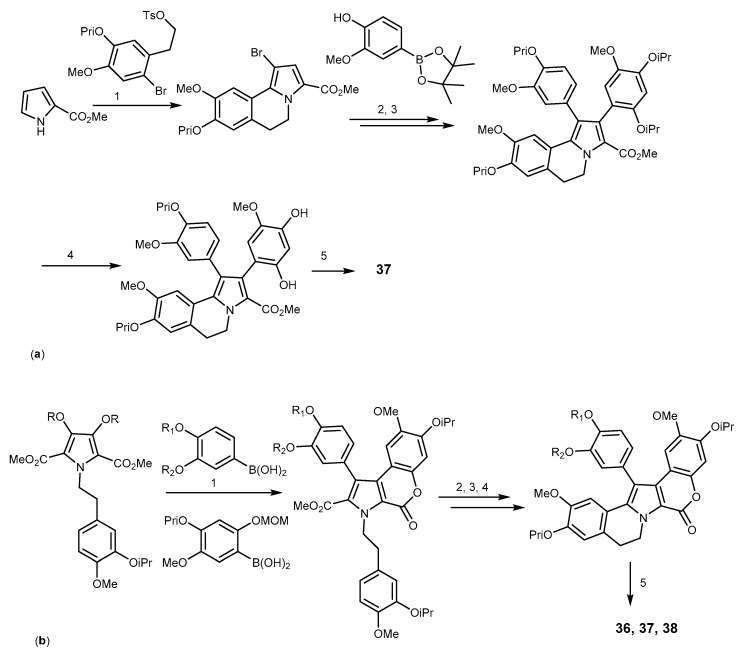
Synthesis of lamellarin D (**37**). *Reagents and conditions*: (**a**) (1) PdCl_2_(PPh_3_)_2_, PPh_3_, K_2_CO_3_, NaH, DMF; (2) PdCl_2_(PPh_3_)_2_, K_2_CO_3_, DMF; (3) NBS, THF; (4) DDQ, CHCl_3_, MW, AlCl_3_, CH_2_Cl_2_; (5) NaH, THF; (**b**) (1) Pd(PPh_3_)_4_, THF, reflux; (2) KOH-EtOH, reflux, then *p*-TsOH, CH_2_Cl_2_, reflux; (3) Cu_2_O, quinolone; (4) Pd(OAc)_2_, CH_3_CN, reflux; (5) BCl_3_, CH_2_Cl_2_.

Finally, cleavage of isopropyl esters, followed by acidic lactonization, afforded **37** in 3% yield, over 16 steps (simplified in Scheme 8a) [109]. In the same year, Pla et al. reported a sequential and regioselective bromination/Suzuki cross-coupling reaction to introduce the aryl group at position 1 of methyl 5,6-dihydropyrrolo[2,1-*a*]isoquinoline-2-carboylate, with the combination of microwave-assisted 2,3-dichloro-5,6-dicynobenzoquinone (DDQ) oxidation, followed by phenol deprotection and lactonization to give **37** in the 18% yield in 8 steps reaction [109]. Iwao et al. also reported the synthesis of **36**, **37**, and **40** in 19%, 18%, and 16% yields over 11, 12, and 12 steps, respectively. They started from isopropyl-protected isovanillin and applied a nitroaldol condensation, a *N*-dialkylation and Hinsberg pyrrole cyclization reactions (Scheme 8b) [110].

**Scheme 9:**
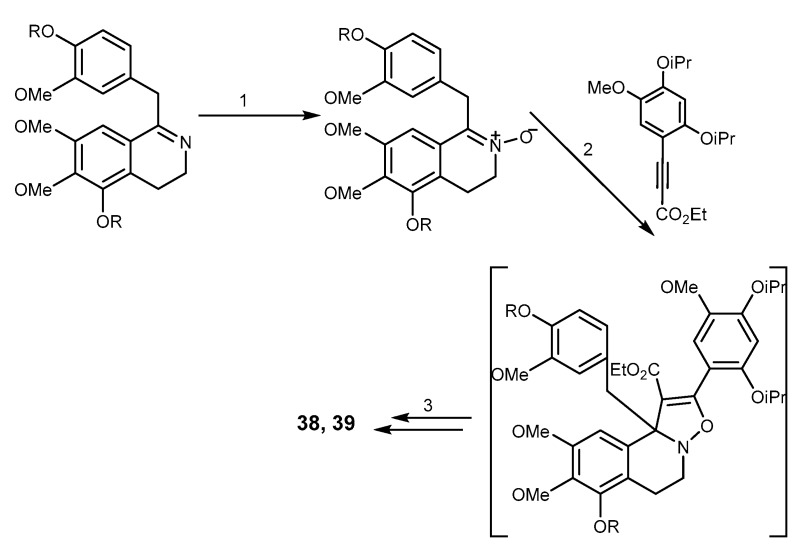
Synthesis of lamellarin I (**38**) and K (**39**) via 1,3-dipolar cyclization of nitrones. *Reagents and conditions*: (1) NaBH_4_, MeOH, H_2_O_2_, Na_2_WO_3_, MeOH; (2) toluene, 120 °C, 18 h; (3) AlCl_3_.

In 2001, Diaz et al. [101] constructed the pyrrole core in [3 + 2]-cycloaddition of nitrone to synthesize **38** and **39** (Scheme 9).

The *N*-oxides were prepared in moderate yields by reduction of 3,4-dihydro-1-benzylisoquinolines, followed by disodium tungstate-catalyzed oxidation with hydrogen peroxide. Reaction of *N*-oxide with alkyne produced an intermediate through the 1,3-dipolar cycloaddition-thermal rearrangement. Selective isopropyl group removal from the intermediate, with concomitant lactonization, gave lamellarins **38** and **39** in 4 and 6% yields, respectively [101]. In 2014, Yamaguchi et al. reported the synthesis of lamellarin I (**38**) using β-selective arylation of pyrroles with aryl iodides and a new double C-H/C-H coupling as a key step to afford **38** in 3% over 8 steps, starting from 2,3,4-trimethoxybenzaldehyde [111].

#### 2.4.2. Ningalins and Derivatives

**Figure 9:**
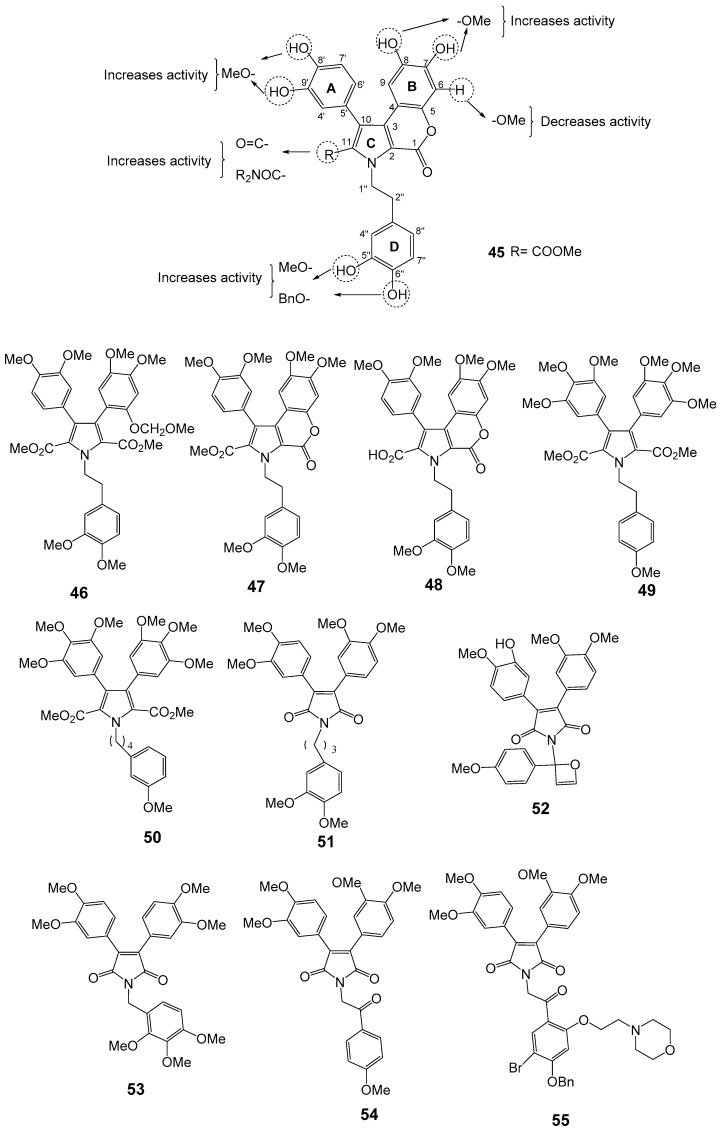
Structures of ningalin B (**45**), analogs **46**–**55**, and SAR studies for P-gp modulation.

Ningalins are members of 3,4-dihydroxyphenyalanine (DOPA)-derived *o*-catechol metabolites including the tunichromes [112]. Biological activities of ningalins include HIV-1 integrase inhibition [113], Ningalin B (**45**, Figure 9), was isolated from an ascidian of the genus *Didemnun* by Boger et al. in 1999. Compound **45** lacks of an intrinsic cytotoxicity which makes this alkaloid an interesting model as MDR reversal agent. Interestingly, the synthetic analogs **46**–**50** were shown to significantly sensitize the HCT116/VM46 human colorectal carcinoma cell overexpressing P-gp to vinblastine and doxorubicin better than the marine natural product **45** and verapamil [114]. Studies of the *N*-aryl homologs of **45** (**50–51**) revealed that increasing the carbon linker leads to increase in activity. The authors concluded that the *N*-alkylaryl substituent is necessary for activity, but the analogs which bear free phenolic groups and methyl esters are more cytotoxic and lack potent MDR reversal properties. Analogs of **45** containing tetra- or pentasubstituted pyrroles showed the highest activity as MDR reversal agents [114,115,116] whereas those having 2-carboxylate or 2,5-carboxylates bearing 3,4-diaryl-substituted pyrroles showed reversal ability toward HCT116/VM46 cells. Compound **51** exhibited the greatest potential in reversing the MDR at 1 µM, and at 10 µM with about a 4000-fold increase in sensitivity against the vinblastine resistant MDR-leukemia cell line. This compound was also shown to compete with [^3^H]-azidopine for P-gp binding site and increased the intracellular accumulation and retention of MDR substrates. In nude mice-xenograft models, the combination of sub-optimal dose of paclitaxel with **51** not only leads to shrinkage the HCT116 tumor size but also to a complete therapeutic remission without increasing toxicity toward the host [116]. The analog **48** showed little or no effect of MDR on HCT116/VM46. The amide analogs of **48** enhanced the MDR activity, while amine derivatives (dimers) were inactive as MDR reversal compounds [115]. The synthetic analog of **46**, 1-[2-(4-methoxyphenyl)-2-oxoetyl]-3,4-bis(3,4-dimethoxyphenyl)-1*H*-pyrrole-2,5-dione (**52**) which was designed from permethyl ningalin B showed a remarkable enhancement of MDR reversal abilities in concentrations up to 1 μM [117,118]. Compound **53**, having a methoxy group on the D-ring, showed a 18.2-fold increase in cell sensitization towards paclitaxel at 1 μM concentration, and a 66 fold one when 0.5 μM of **53** was combined with 0.5 μM of **54** [117]. It was found that increasing the number of methoxy groups on ring B of the ningalin scaffold showed only low to moderate P-gp-modulating activity, while the addition of a benzyloxy group on the D ring enhanced the P-gp modulating activity [119]. SAR studies of a series of novel *N*-substituted derivatives of **45** on breast P-gp-overexpressing tumor cell line (LCC6MDR) showed that analogs containing one methoxy group and one benzyloxy group on ring C are the most potent P-gp modulators (1 μM desensitized cell line by 42.7 folds) without showing cytotoxicity IC_50_ for L929 fibroblast >100 μM) [117,119,120]. The analog **55** containing dimethoxy groups on rings A and B, and trisubstitution with *o*-methoxyethylmorpholine, *m*-bromo and *p*-benzyloxyl on ring D showed an effect compatible with P-gp modulation with an effective concentration (EC_50_) of 423 μM in reversing paclitaxel resistance [118]. Figure 9 summarizes the SAR established for this class of compounds against P-gp modulation highlighting the most promising ningalin derivatives.

**Scheme 10:**
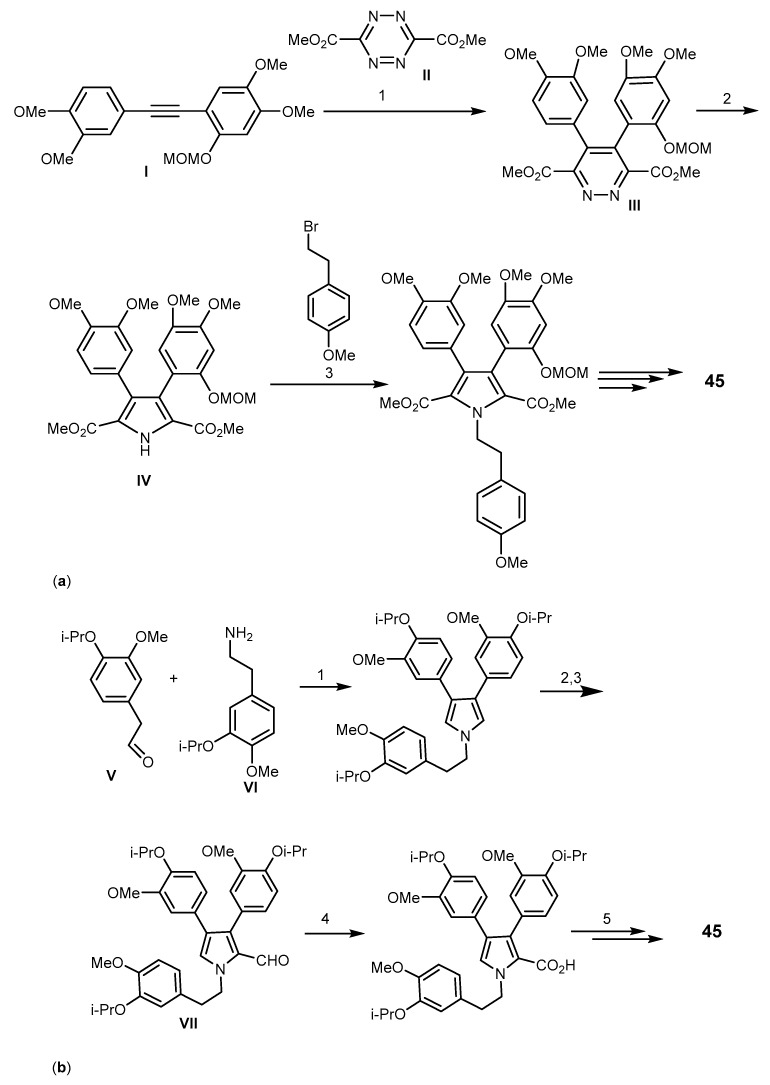
Synthesis of ningalin B (**45**). *Reagents and conditions*: (**a**) (1) N_2_; (2) Zn, AcOH; (3) K_2_CO_3_; (**b**) (1) AgOAc, NaOAc, THF; (2) POCl_3_, DMF; (3) DMSO/H_2_O or THF: *t*-BuOH:H_2_O; (4) Pb(OAc)_4_, EtOAc; (5) BBr_3_, CH_2_Cl_2_.

The synthesis of ningalin B (**45**) was described by Boger et al. using the Diels-Alder reaction of the acetylene **I** with 1,2,4,5-tetrazine **II** to give the 1,2-diazine **III**. The synthetic pathway includes the transformation of 1,2-diazine **III** to the pyrrole **IV**, followed by *N*-alkylation, lactonization and decarboxylation, and demethylation to yield **45** (Scheme 10a) [121,122]. Another synthesis of ningalin B (**45**) started from the aldehyde **V** and the amine **VI**, as depicted in Scheme 10b, and was accomplished by oxidative coupling reaction to yield the pyrrole moiety, followed by Vilsmeier-Haack formylation under traditional heating to give the formylpyrrole **VII** which was subsequently converted to the lactone and then to ningalin B (**45**) [113].

#### 2.4.3. Welwitindolinones and Derivatives

**Figure 10:**
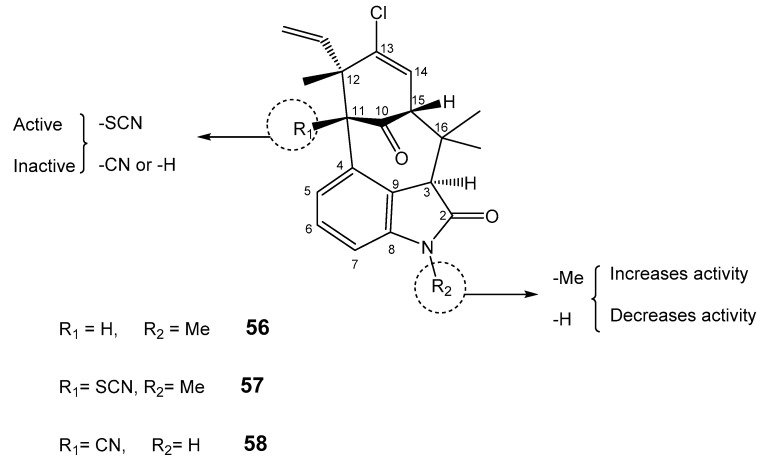
Structures of welwitindolinones **56**–**58** and SAR studies. The dashed circle indicates the groups important for P-gp.

Welwitindolinones are alkaloids found in the cyanobacteria *Hapalosiphon weltischii*, *Westiella intracta*, *Fischerella muscicola,* and *F. major* [123,124]. The extracts which significantly contain *N*-methylwelwitindolinone (**56**) were found to have potent biological activities including antifungal, larvicidal, and insecticidal properties [124]. Most welwitindolinones possess a densely functionalized oxindole-fused bicyclo[4,3,1]decane ring system (Figure 10). The core structure of these alkaloids contains two contiguous stereogenic centers. These alkaloids exhibit a significant activity in reversing P-gp-mediated drug resistance in human tumor cells [125]. Among them, *N*-methylwelwitindolinone C (**56**) enhanced cytotoxicity of anticancer drugs like vinblastine, taxol, actinomycin D, colchicine, and daunomycin in MDR breast carcinoma cell line. The absence of the *N*-methyl group reduced the activity, while the absence of the isothiocyanate group completely abolished the activity, as observed for welwitindolinone C isothiocyanate (**57**) and welwitindolinone isonitrile (**58**), respectively. Welwitindolinone **57** was found to be the most potent derivative in increasing the accumulation of [^3^H]-vinblastine and [^3^H]-paclitaxel by blocking P-gp in SK-VLB-1 cells. This compound also inhibits the P-gp photoaffinity labeling by [^3^H]-azidopine in MDR cells [36].

**Scheme 11:**
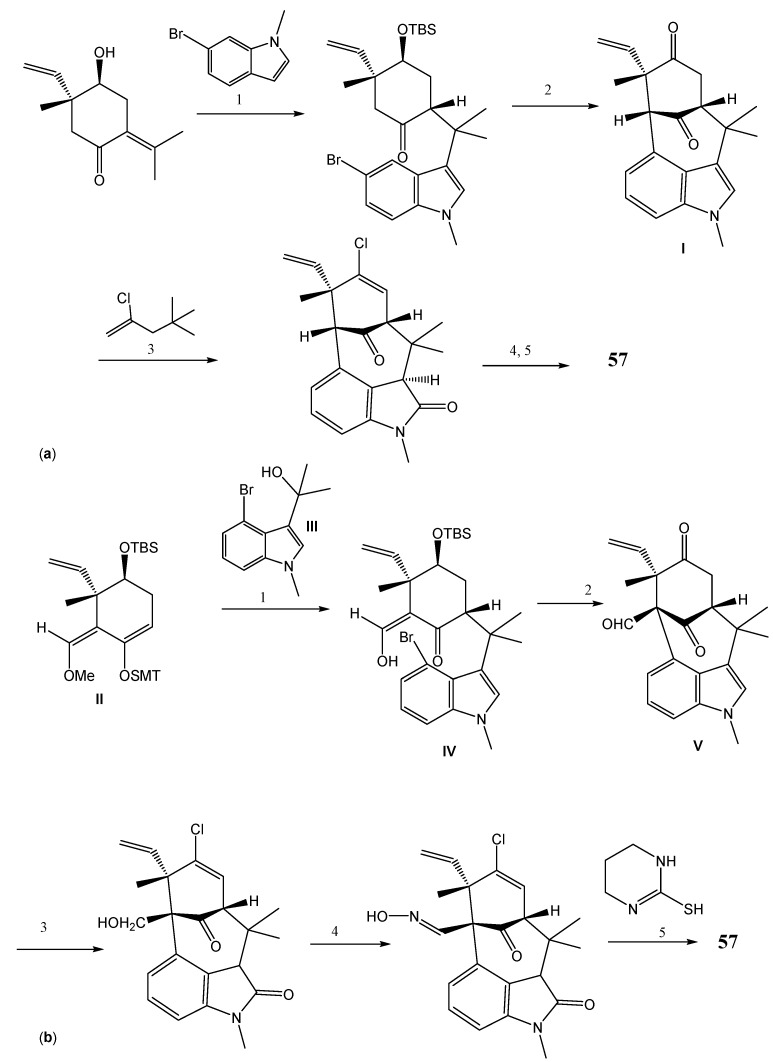
Synthesis of *N*-methylwelwitindolinone C isothiocyanate (**57**) by Gang’s approach (**a**) and Rawal’s approach (**b**). *Reagents and conditions*: (**a**) (1) iodine promoted bromination; (2) NaNH_2_, *t*-BuOH, THF; (3) trimethyethylstannane; (4) LiEt_3_B-D, THF, Cl_3_CCONCO, CH_2_Cl_2_, K_2_CO_3_, MeOH; AgOTf, PhI(OAc)_2_, CH_3_CN, bathophenantroline; (5) NaH, air, THF; (**b**) (1) TiCl_4_, toluene; (2) Pd(OAc)_2_, P-*t*Bu_3_, KO-*t*Bu, toluene; (3) NaBH(OMe)_3_, THF/EtOH then N_2_H_2_, AcOH, EtOH; NCS, pyridine; MMPP, TFA, AcOH; (4) Dess-Martin periodinane, NaHCO_3_, CH_2_Cl_2_; NH_2_OH.HCl, pyridine, MeOH; (5) NCS, DMF, THF, then Et_3_N.

Compound **57** was synthesized by two different approaches—Gang’s and Rawal’s. In Gang’s method, the synthetic pathway begins with coupling of a functional cyclohexanone using iodine promoted bromoindole addition, followed by ring closure to form the key intermediate compound **I**. Thereafter, **57** was formed by chlorination through vinyltrimethylstannane oxidation to oxindole, isotopically enhanced tethered nitrene insertion, and isothiocyanate introduction (Scheme 11a) [126,127,128]. In Rawal’s approach (Scheme 11b), the coupling of silylenolether **II** with nucleophile bromoindole **III** was achieved by Lewis acid to form the corresponding cyclohexanone **IV** which then produces the key intermediate **V** by palladium-catalyzed enolate arylation [124,129]. The key intermediate compound **V** was subjected to aldehyde reduction via a hydrazine (oxidation to the oxindole), alcohol oxidation and oxime formation, and Curtius rearrangement of oxime to isothiocynate to furnish **57**.

#### 2.4.4. Harmine and Derivatives

**Figure 11:**
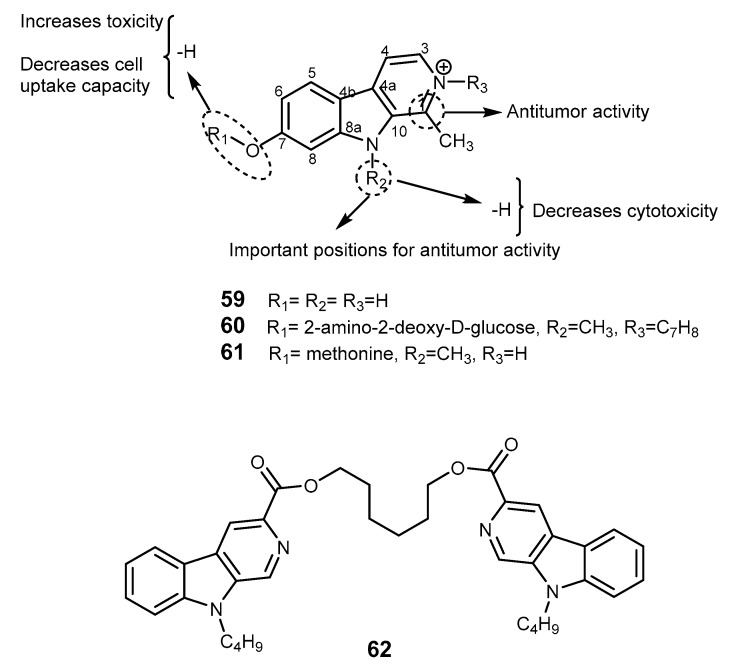
Harmine (**59**) and analogs **60**–**62**. The dashed circles indicate groups important for biological functions.

Harmine (**59**) is a β-carboline alkaloid found in plants, mammalians, insects, and marine organisms [130], e.g., the marine brown alga *Melanothamnus afaqhusainii* [131,132]. The plant extracts containing **59** were traditionally used to treat alimentary tract cancer and malaria in Northwest China [133]. These extracts have a broad spectrum of biological activities, including antimicrobial, antifungal, antitumor, antiplasmodial, antioxidant, antimutagenic, antigenotoxic, as well as hallucinogenic properties [130]. Compound **59** also inhibits topoisomerase I, cyclin-dependent kinases, monoamine oxidase A, and intercalates into DNA [134]. Studies on the biological function of **59** reported this alkaloid as a potent antiproliferative and cytotoxic agent, with MDR reversal activity by inhibiting BCRP in a BCRP overexpressing breast cancer cell line (MDA-MB-123) and by reducing the resistance of mitoxantrone and camptothecin mediated by BCRP, but did not inhibit *MDR-1* overexpressing cells growth [135]. More detailed studies of **59** revealed that this compound failed clinical in applications due to its low pharmacological effects and neurotoxicity [132,134]. To improve the therapeutic efficacy of **59**, several derivatives were synthesized by modification of substituents in positions 2, 7, and 9 of harmine (**59**) ring and with other two different groups—2-amino-2-deoxy-d-glucose and methionine—two derivatives were obtained, 2DG-Har-01 (**60**) and MET-Har-02 (**61**), respectively. This study found that substitution at position 7 reduced natural toxicity and increased tumor cell uptake ability, at position 9 enhanced cytotoxicity, and at position 2 enhanced the antiproliferative effect [132], and also showed that substituents at position 1 could be crucial to antitumor potency [136]. SAR studies confirmed that substituents in position 2 and 9 displayed an important role in modulation of antitumor activities [134]. Derivatives of **59** that possess in R_1_, R_2_, and, R_3_ (Figure 11) a benzyl and 3’-fluorobenzyl groups are more likely to act as protein synthesis inhibitors [137].

The scaffold of harmine (**59**, β-carboline) was a model for the synthesis of novel compounds which are bivalent β-carbolines. The different number of methylene units in the linker between two β-carbolines produced agents which displayed good and selective cytotoxicity against 769-P and KB cell lines, being the six methylene unit derivative **62** a potent antitumor compound against Lewis lung cancer in mice [136].

The synthesis of β-carboline-based alkaloids has been reported using tryptophan as starting material [138,139]. To the best of our knowledge, there is no report on the total synthesis of **59**. Most of the harmine analogs were synthesized from the harmine scaffold using the Friedel-Crafts reaction, molecular modifications, and *N*-oxidation [132,140,141,142].

#### 2.4.5. Indolcarbazoles and Derivatives

**Figure 12:**
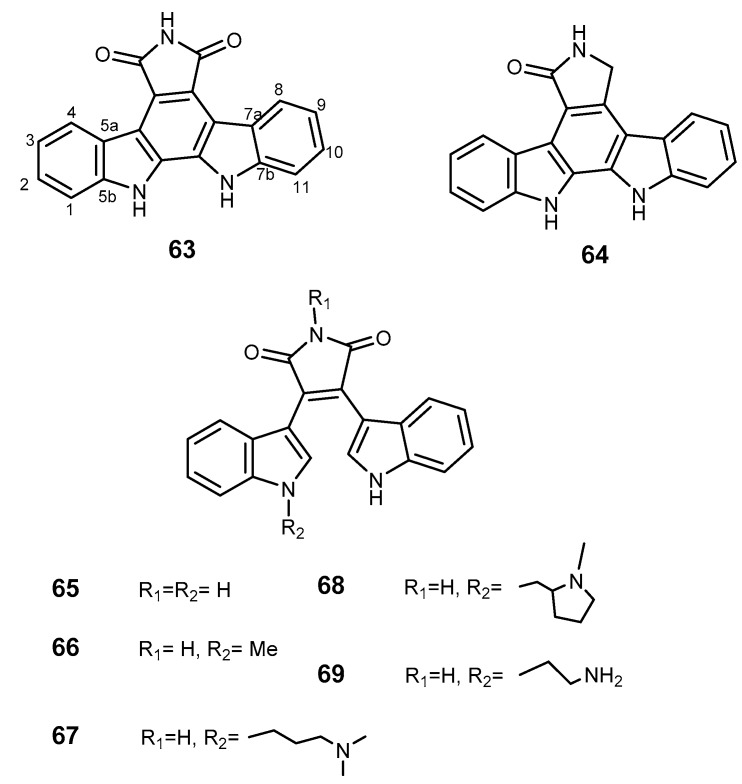
Structures of indolcarbazole derivatives **63**–**69**.

Indolcarbazoles are alkaloids isolated from marine-derived actinomycetes strain Z039-2 which possess a broad spectrum of biological activities including anticancer activity [143]. The mechanisms of their antitumor effects include topoisomerase I poisoning, inhibition of PKC, PKA, pyruvate dehydrogenase kinase (PDK)/cyclin B, and cyclin-dependent kinase 5 (CDK5)/p5 [144,145]. The naturally occurring arcyriaflavin (**63**) and indolcarbazole K252c or staurosporin aglycone (**64**, Figure 12) showed the most potent effects in BCRP inhibition in the BCRP-transferred HEK-293 cell line, with low toxicity in BCRP-transfected cells, and reduced the relative resistance of ABCG2-transfected cells SN-38 [29,146]. Bisindolylmaleimide analogs **65**–**69** (Figure 12) were found to be able to inhibit [^125^I]iodoarylazidoprazosin labeling of BCRP by 65% to 80% at 20 μM [146]. These findings revealed that indolocarbazole and bisindolylmaleimide analogs directly interact with BCRP protein and may increase oral bioavailability of BCRP substrates.

**Scheme 12:**
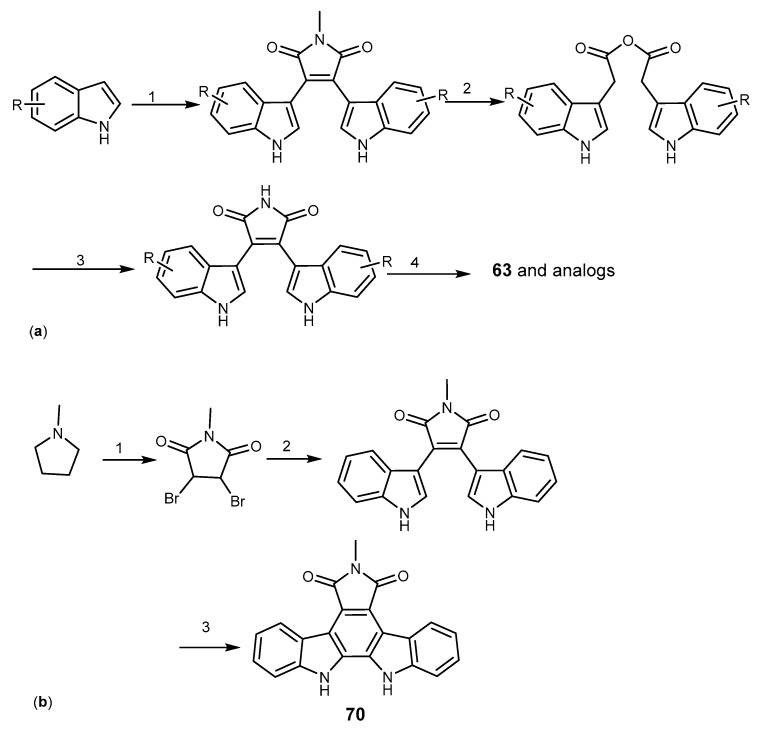
Synthesis of indolcarbazole derivatives **63** and **70**. *Reagents and conditions*: (**a**) (1) i. EtMgBr, THF, benzene, ii. *N*-methylmaleimide; (2) KOH, MeOH or dioxane; (3) NH_4_OAc; (4) DDQ, PTSA, toluene; (**b**) (1) Br_2_, HNO_3_; (2) CH_3_CH_2_Br, Mg, THF, indole, toluene; (3) DDQ, toluene.

The indolocabazole nucleus was prepared from bis-indolylmaleimides, followed an oxidative cyclisation to give the indolocarbazole (Scheme 12a). Another way to obtain the indolocarbazole nucleus was applied in the preparation of *N*-methyl indolocarbazole (**70**) which was obtained from *N*-methylpyrrole by bromination and oxidation to give *N*-methyldibromomaleimide which was coupled with indolylmagnesium bromide to afford bis-indolylmaleimide. Oxidative cyclization of the later compound led to indolocarbazole **70**, as depicted in Scheme 12b [145,147,148].

#### 2.4.6. Ecteinascidin 743 (Trabectedin) and Derivatives

**Figure 13:**
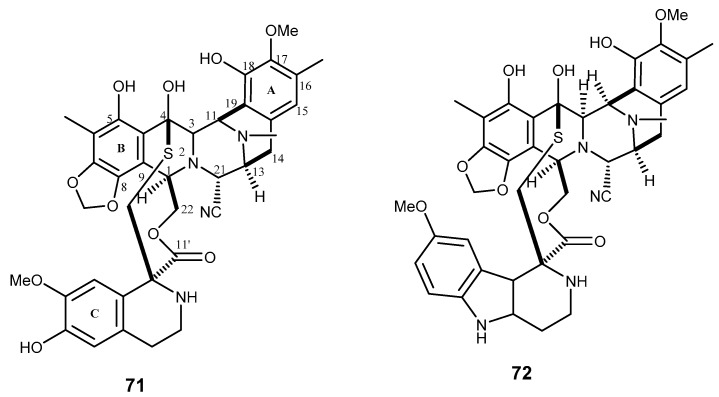
Structures of trabectedin (**71**) and lurbinectedin (**72**).

Esteinascidin 743 or trabectedin (**71**, Figure 13) is an anticancer tetrahydroisoquinolone alkaloid already approved by the FDA. This compound was first isolated from the Caribbean tunicate *Ecteinascidia turbinata* in a minute quantity. However, this compound could be synthesized from cyanosafracin B, a starting material obtained from fermentation of *Pseudomonas fluorescens* [149]. It is a highly potent anticancer agent and has activity against a number of human solid tumor cells. The compound was also showed to reverse the resistance to doxorubicin and vincristine in KB-C2 and KB-8-5 cells overexpressing P-gp in concentrations of 0.1 nM [150,151]. Trabectedin (**71**) was approved as the medicine Yondelis^®^ for the treatment of advanced soft-tissue sarcomas and ovarian cancer. Although **71** is a substrate for P-gp, this potential mechanism of resistance has only been shown to be significant at concentrations exceeding clinically relevant values [151]. Moreover, this marine natural product also inhibits the activation of the *MDR-1* gene that encodes for P-gp [152]. Therefore, **71** could be considered a promising model to overcome MDR. For instance, the synthetic agent lurbinectedin or PM01183 (**72**), which was obtained by modification of the subunit C of **71**, showed a potent cytotoxic activity against tumor cell line of different origin, and it was introduced in phase I clinical trials for solid tumor, and phase II for metastatic pancreatic cancer. PM01183 (**72**) showed enhanced activity to cisplatin- and oxaliplatin-resistant cell line, and combination of **72** and cisplatin was the most synergistic toward parental and cisplatin-resistant ovarian carcinoma cells [153].

**Scheme 13:**
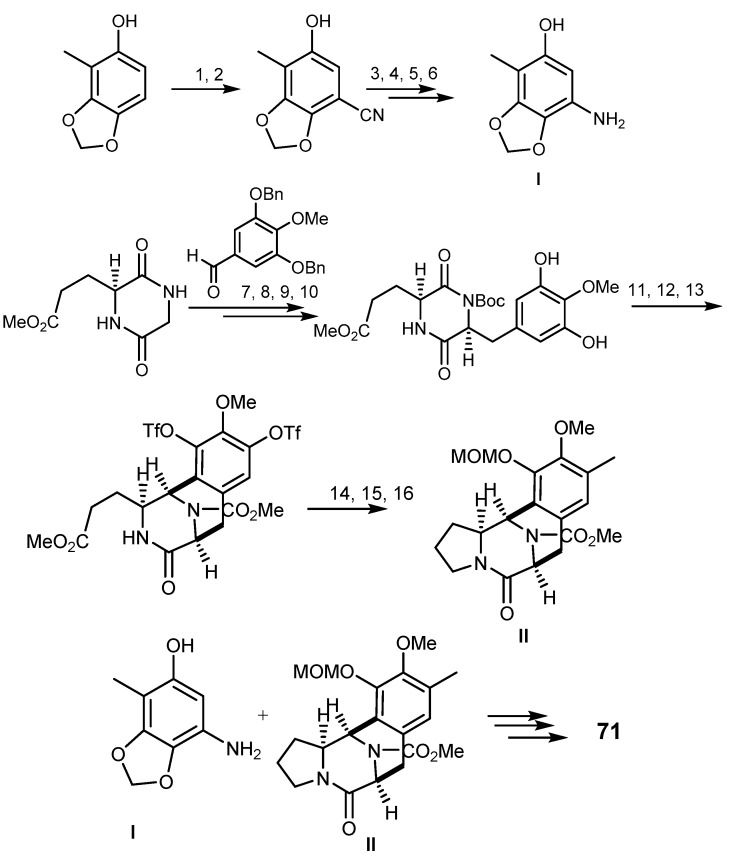
Total synthesis of trabectedin (**71**). *Reagents and conditions*: (1) PhI(OAc)_2_, MeOH; (2) NaCN, DMF/H_2_O; (3) BnBr, K_2_CO_3_, DMF; (4) aq H_2_O_2_, K_2_CO_3_, DMSO; (5) PhI(OAc)_2_, KOH, MeOH; (6) LiOH, EtOH/H_2_O, reflux; (7) *t*-BuOK, THF, DBU, (8) Boc_2_O, DMAP, THF, (2 steps); (9) H_2_ (750 psi), Pd/C, EtOAc; (10) H_2_NNH_2·_H_2_O, THF, evaporation; NaBH_4_, MeOH, (2 steps); (11) TFA, CF_3_CH_2_OH; evaporation; PhNTf_2_, DMAP, Cs_2_CO_3_, MeCN; (12) trimethylboroxine, Pd(PPh_3_)_4_, K_3_PO_4_, 1,4-dioxane; (13) HCl, EtOAc; ClCO_2_Me, NaHCO_3_, H_2_O; (14) l-Selectride, THF; (15) CSA, toluene, reflux (2 steps); (16) aq KOH, 1,4-dioxane, rt, MOMCl.

Syntheses of trabectedin (**71**) have been described by many researchers. In summary, there are three important pathways (Scheme 13): (A) combination of two fragments (**I** and **II**) via an oxazolidine intermediate, (B) acid-promoted macrocyclization via creation of carbon-sulfur bond with concomitant formation of a 10-membered ring, and (C) the intramolecular Pictet-Spengler reaction to obtain **71** [154,155,156,157]. The straightforward synthesis of **71**, using 28 steps with 1.1% yield, started from l-glutamic acid as the single chiral source [158] while a 31 steps synthesis, with 1.7% yield, began from 3-methylcatechol [159]. The first conditions lead to the B-ring formation by stereoselective Heck reaction between diazonium salt and enamide, oxidative cleavage of the resulting alkane and intramolecular *ortho* substitution of phenol by aldehyde. This pathway can form a diketopiperazine by Perkin condensation and the bicyclo[3.3.3]system by an *N*-acyliminium ion-mediated cyclization, and a regioselective Suzuki-Miyaura coupling (Scheme 13) [158]. In 2000, Cuevas et al. [149] established the synthesis of **71** and **72** from cyanosafracin B, which is an antibiotic obtained from fermentation of the bacterium *Pseudomonas fluorescens*. The methoxy-*p*-quinone of cyanosafracin B was treated with bromochloromethane, followed by Edman degradation to form thiourea (Scheme 13). The critical substitution of the amino group by an alcohol group gave the key intermediate to yield **71** through the Corey method [149].

### 2.5. Diketopiperazines

#### 2.5.1. Nocardioazines

**Figure 14:**
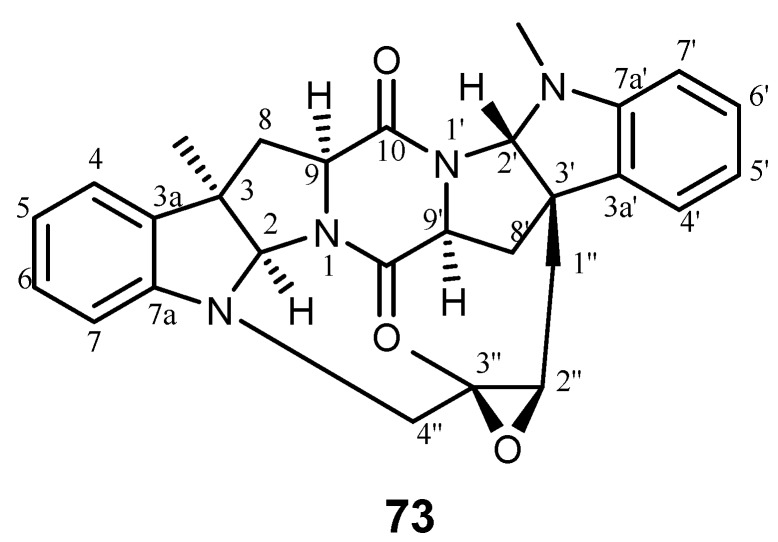
Structure of nocardioazine A (**73**).

Nocardioazines are bridged diketopiperazine alkaloids which were isolated from a non-saline liquid culture of *Nocardiopsis* sp. (CMB-M0232). Nocardioazine A (**73**) is composed of cyclo-(l-Trp-l-Trp) and cyclo-(l-Trp-d-Trp), as shown in Figure 14. This compound has revealed an effect compatible with P-gp inhibition in a P-gp overexpressing colon cancer cell line (SW60Ad300) [160].

**Scheme 14:**
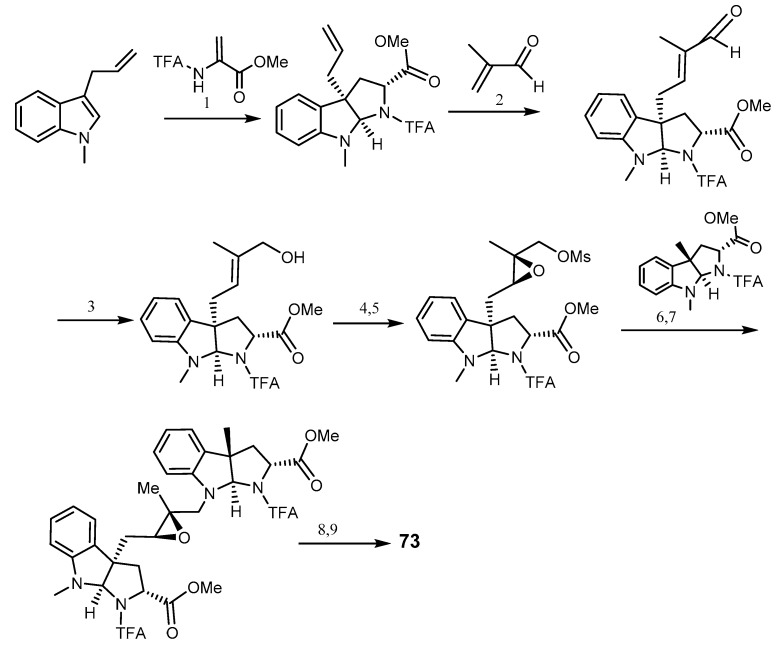
Total synthesis of nocardioazine A (**73**). *Reagents and conditions*: (1) (*S*)-BINOL, SnCl_4_, DCM; (2) *O^i^*Pr-HG II, DCM, reflux; (3) NaBH_4_, CeCl_3_·7H_2_O, MeOH; (4) (+)-diethyl tartrate, Ti(O*^i^*Pr)_4_, tBuOOH, 4Å MS, DMC; (5) MsCl, Et_3_N, THF; (6) TBAI, DIPEA, CH_3_CN; (7) Pd_2_(dba)_3_, dppb, DMBA, DEC; (8) LiOH, THF/H_2_O; (9) PyBrop, DIPEA, DMF.

The first synthesis of (+)-nocardioazine A (**73**) was reported in 2014 by Wang and Reisman. The synthetic pathway comprises the building of the pyrroloindoline unit from an enantioselective formal [3 + 2] cycloaddition, and an unusual intramolecular diketopiperazine formation. The synthesis was performed from 3-allylindole in nine steps which gave an overall yield of 11% of **73** (Scheme 14) [161].

#### 2.5.2. Fumitremorgins and Derivatives

**Figure 15:**
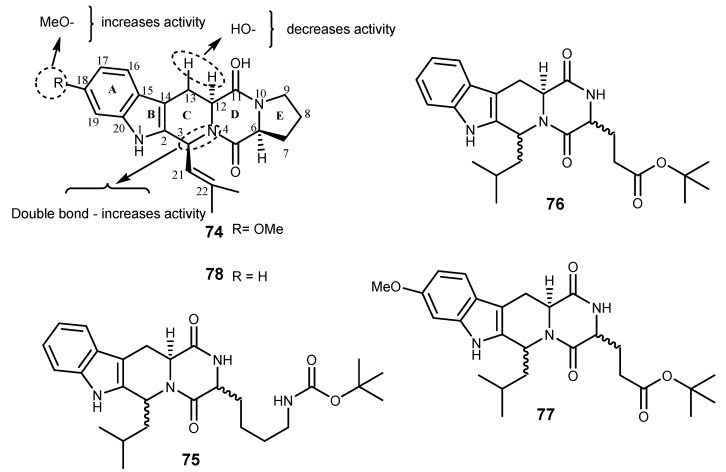
Structures of fumitremorgin C (**74**) and derivatives **75**–**78** and SAR for BCRP inhibition.

Fumitremorgin C (FTC, **74**) is an indolyl diketopiperazine alkaloid found in several marine fungi such as the fungal strain BM939, fungal A-f-11, *Aspergillus sydowii*, and *Aspergillus fumigatus* (YK-7) [162]. Fumitremorgins were found to be tremorgenic mycotoxins by interfering the releasing neurotransmitters and also showed inhibitory activity on cell cycle [163]. Compound **74** was reported as a chemosensitizing agent that could reverse a drug-resistant cell lines that do not overexpress P-gp and MRP [163,164]. This effect was shown to be due to BCRP expression in the S1-M1-3.2 cell line resistant to mitoxantrone, topotecan, and doxorubicin [164]. Fumitremorgin C (**74**) almost completely reversed resistance mediated by BCRP in MCF-7 cells transfected with this protein [165]. Moreover, **75** was found to inhibit nitrofurantoin and mitoxantrone (BCRP’s substrates) transcellular transport in MDCKII and MEF3.8 cell lines [166]. The SAR study using the *ftm* gene cluster revealed that the moieties that are essential for inhibitory activity of **74** against BCRP are the double bond between C-3 and N-4, as well as the methoxy group at C-18, however, the hydroxyl groups at C-12 and C-13 lead to a decrease in activity (Figure 15) [167]. Unfortunately, **74** was found to induce tremors or convulsion in mice and other animals [168]. Two derivatives, Ko132 (**75**) and Ko134 (**76**), were found to be potent inhibitors of the BCRP-mediated drug efflux in T6400 mouse and T8 human cell lines with low cytotoxicity at an effective concentration of 1 μM [163,169]. In addition, **77** was the most effective inhibitor of BCRP, with very low activity against P-gp or other known drug transporters [168,170]. In addition, the stereospecificity was shown to be very critical in the Ko family, for example, compounds having the 3*S*,6*S*,12α*S* configuration were 18 times more potent than those with 3*S*,6*R*,12α*S* configuration in inhibiting BCRP; however, this stereoselective effect was not observed in P-gp and MRP-1 [171].

**Scheme 15:**
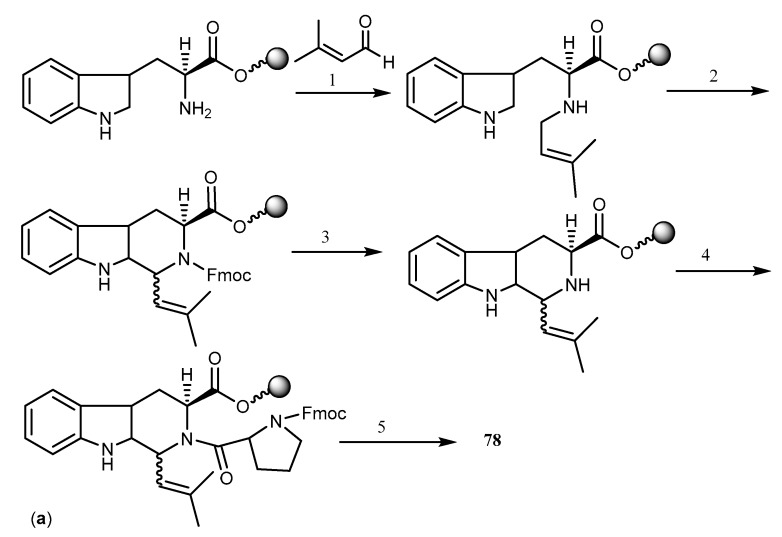

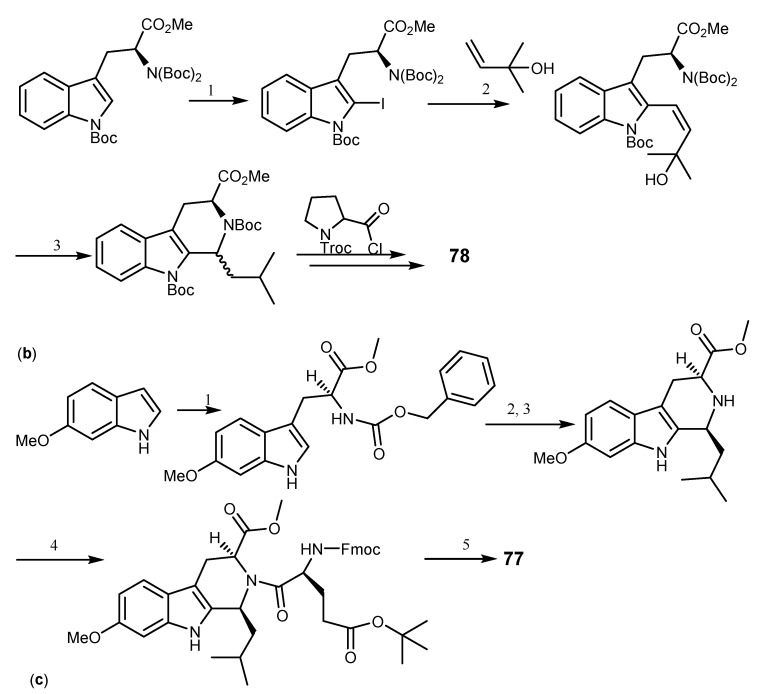
Total synthesis of FTC derivatives, dimethoxy-FTC (**78**) and Ko143 (**77**). *Reagents and conditions*: (**a**) (1) CH(OMe)_3_; (2) Fmoc-HCl, pyridine, CH_2_Cl_2_; (3) piperidine, DMF; (4) Fmoc-l-pro-OH, CIP, DiPEA, MNP; (5) piperidine, THF; (**b**) (1) Hg(OOCCF_3_)_2_, KI, I_2_, CH_2_Cl_2_, then Boc_2_O, DMAP, MeCN; (2) Pd(OAc)_2_, Ag_2_CO_3_, toluene; (3) Mg(ClO_4_)_2_, MeCN; (**c**) (1) 1-benzyl-2-methyl-(*S*)-1,2-aziridinedicarboxylate, ytterbium triflate, CH_2_Cl_2_; (2) H_2_ balloon, MeOH, 10% Pd/C; (3) isovaleraldehyde, TFA, CH_2_Cl_2_; (4) *N*-Fmoc-5-*t*-butyl l-glutamic acid ester, diisopropylethylamine 2-chloro-1,3-dimethylimmidazolinium hexaflorophosphate, *N*-methylpyrrolidinone; (5) piperidine, THF.

The first synthesis of **74** was reported by Hino et al. who prepared *N*-propyl-7-methoxy-β-carboline as the key intermediate [172]. Loevezijn et al. reported a solid phase synthesis of demethoxy-FTC (**78**), also an inhibitor of BCRP-mediated MDR, by a cyclization/cleavage multiple parallel syntheses method (Scheme 15a). This synthetic pathway was based on the formation of the diketopiperazine rings system, followed by simultaneous cleavage of solid support, which acted as a leaving group at the cyclization step. The reaction was performed by the Pictet-Spengler condensation of the hydroxyethyl functionalized polystyrene resin linked l-tryptophan with excess of aldehyde Scheme 15a [163,169].

Another alternative to obtain **78** was achieved by Mg(ClO_4_)_2_-catalyzed intramolecular allylic amination with carbamate or sulfonamide as nucleophiles to form substituted piperidine and pyrrolidine (Scheme 15b) [173]. The synthesis of Ko143 (**77**), a potent BCRP inhibitor of the FTC series was accomplished and optimized by Yuexian et al. [174] which involved ytterbium triflate-promoted coupling between 6-methoxyindole and optically active 1-benzyl-2-methyl-(*S*)-1,2-aziridinedicarboxylate (Scheme 15c) [174].

#### 2.5.3. Halimide and Derivatives

**Figure 16:**
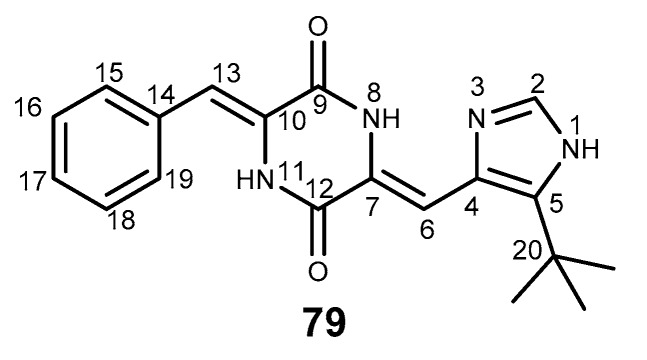
Structure of plinabulin (**79**).

Halimide, is a diketopiperazine secondary metabolite isolated from the marine fungus *Aspergillus ustus.* This compound exhibited potential in vitro cytotoxic activity against human colon and ovarian carcinoma cells [175]. Structurally, halimide is composed of a phenylalanine, a histidine, and a tertiary butyl group. An analog of halimide, plinabulin (KPU-2, **79**, Figure 16), that was obtained by molecular modification at the phenyl ring, has revealed a potent in vitro antitumor activity not only against various human tumor cell lines, but also against those with various MDR profiles [176]. Moreover, **79** was reported to have a similar mechanism of action to that of eribulin (**31**) as microtubule-disrupting agent with colchicine-like tubulin-depolymerizing activity, but the main problem concerning **79** is its poor solubility (<0.1 μM), which can trigger further studies on this scaffold. The phase I/II clinical trial of **79** in combination with docetaxel was completed in patients with advanced non-small cell lung cancer, and compound **79** was shown to act synergistically with docetaxel in murine models of NSCLC [177].

### 2.6. Peptides

#### 2.6.1. Hapalosin and Derivatives

**Figure 17:**
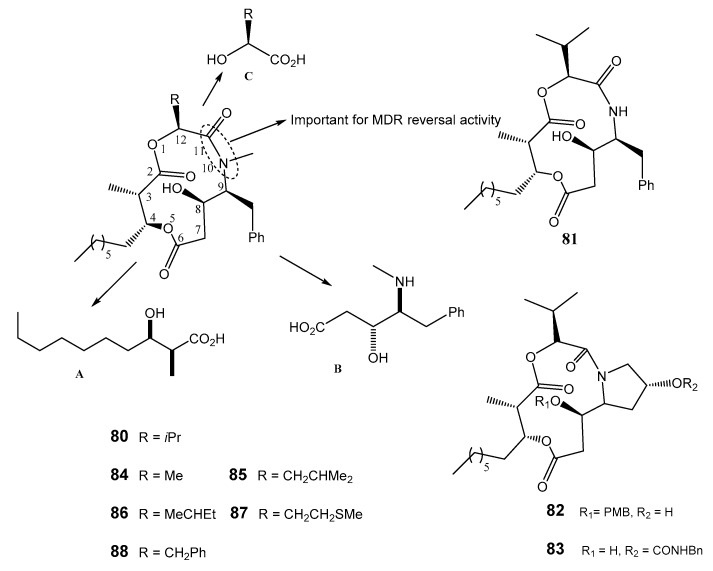
Structure of hapalosin (**80**) and analogs **81**–**88** highlighting the important domains for synthesis (**A**–**C**).

Hapalosin (**80**, Figure 17) is a cyclic depsipeptide, isolated from the lipophilic fraction of the extract of a cyanobacterium *Hapalosiphon welwitschii* W. & G.S West, and was found to reverse MDR in a P-gp overexpressing, vinblastine-resistant human ovarian adenocarcinoma cell line with higher effect than the known P-gp inhibitor verapamil [178,179].

At 20 μM concentration, compound **80** significantly enhanced the accumulation of [^3^H]-paclitaxel in SKVLB1 cells, and exhibited a similar activity to verapamil in breast cancer cell MCF-7/ADR in the range of 1.5–10 μM [180,181]. Several syntheses for **80** and its respective analogs have been reported, with the purpose of obtaining compounds with similar or better MDR reversal activity. Some of the analogs did not fulfill this objective; for example, the glucose mimic of hapalosin (**80**) and 8-deoxyhapalosin, the triamide analogs, and *N*-demethylhapalosin **81** possessing a *trans*-amide, exhibited weak MDR reversal activity [180,181,182]. Through SAR studies, proline-containing congeners (**82** and **83**) were found to be more potent against MCF-7/ADR cells than **81** [182]. Substitution at C-12 in **80** furnished analogs **84**–**88**, which exhibited higher vincristine accumulation than verapamil and **80** in MDR 2780AD cells at 10 μM [183]. It was postulated that the *cis*-peptide might be essential to express the MDR-reversing activity, and the bioactive function of **80** and analogs depends on the *S-cis* or *S-trans* configuration [182,184]. Also, a free hydroxyl and aromatic groups may be important for the anti-MDR activity of hapalosin (**80**) and its analogs.

**Scheme 16:**
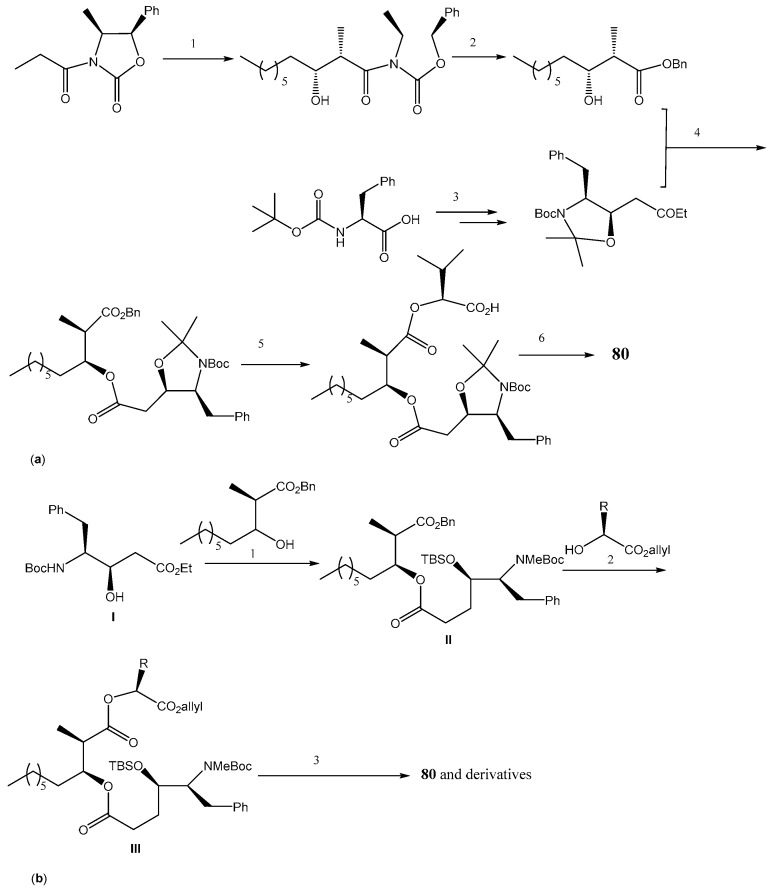
Synthesis of hapalosin (**80**) and derivatives. *Reagents and conditions*: (**a**) (1) Bu_2_BOTf, Et_3_N/CH_2_Cl_2_, then Me(CH_2_)_6_CHO; (2) *n-*BuLi, BnOH/THF; (3) Ref. [193]; (4) NaOH, DCC, DMAP/CH_2_Cl_2_; (5) H_2_, Pd(OH)_2_/EtOH, vinyl-2-hydroxy-3-methylbutanoate, DCC, DMAP/CH_2_Cl_2_; (6) TFA/CH_2_Cl_2_, DPPA, *i*-Pr_2_NEt/DMF; (**b**) (1) i. DCC, DMAP/CH_2_Cl_2_, ii. H_2_, Pd(OH)_2_/EtOH, (2) DCC, DMAP/CH_2_Cl_2_; (3) i. HF-pyridine, ii. (Ph_3_P)_4_Pd, morpholine, then TFA, iii. DPPA, EtN*t*-Pr.

The structure of hapalosin (**80**) was investigated for synthesis, and it was divided into 3 main domains: a β-hydroxy acid (A), a γ-amino-β-hydroxy acid (B), and an α-hydroxy acid (C). Hapalosin (**80**) was initially synthesized by macrolactonization and by cycloamidation [185]. Nobuki et al. reported the synthesis of **80** and analogs by cyclization, producing the peptide bond at the end of the process which allowed obtaining **80** in 44% yield. They used the coupling of *N*-propionyl-(4*S*,5*R*)-4-methyl-5-phenyl-2-oxazolidinone with octanal (Scheme 16a) [181,186]. Another synthesis of hapalosin (**80**) and its analogs was accomplished by a macrolactonization strategy [187,188,189]. The method was based on a chiral acetate reagent, which offered advantages to the aldol reaction and avoided intramolecular cyclization during amino group deprotection and segment coupling [189]. Shigeru et al. presented the synthesis of hapalosin (**80**) and derivatives with modification at C-12 by cyclization producing the peptide bond at the final stage [183]. According to their strategy, hapalosin (**80**) and derivatives were commenced by using γ-amino-β-hydroxy acid **I** which was obtained by either Evans aldol/Curtius combination route [190] or asymmetric dihydroxylation route [191].

Then, the catalytic hydrogenation of the benzyl ester **II** was performed, and the generated carboxylic acid was coupled with an appropriate allyl α-hydroxylate, leading to allyl ester **III**. After sequential deprotection, cyclization produced **80** and derivatives (Scheme 16b). Kumar et al. also described a flexible and highly diastereoselective synthesis of hapalosin (**80**) with the addition of an organometallic reagent to *N-tert*-butanesulfinylimine, the non-aldol aldol reaction, and the Yamaguchi esterification as key steps in this strategy [192].

#### 2.6.2. Botryllamides and Derivatives

**Figure 18:**
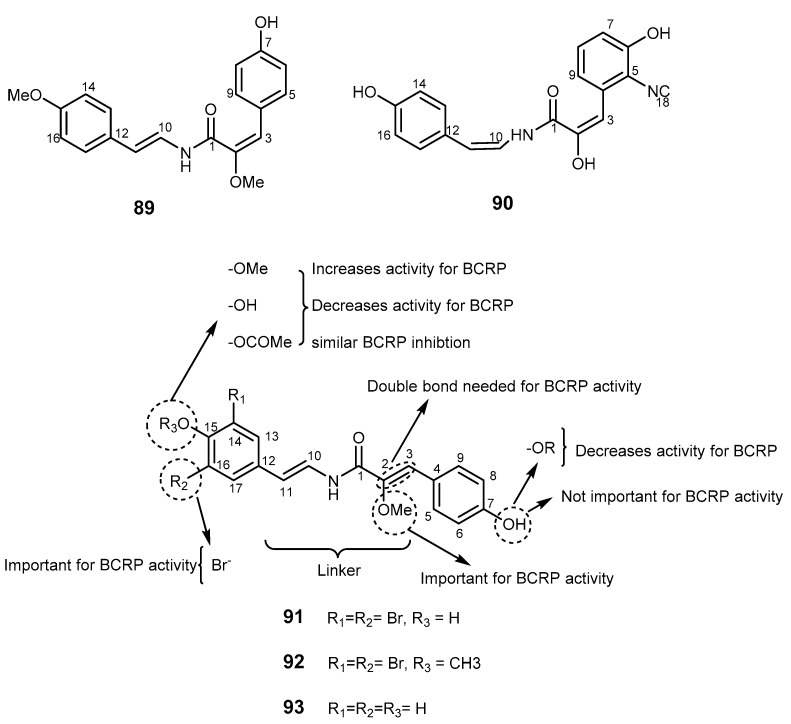
Botryllamides **89**–**93** and SAR studies on BCRP inhibitory activity.

Botryllamides are a group of dehydrotyrosine derivatives, isolated from styelid ascidians *Botryllus* sp. [194,195]. Some botryllamides exhibited weak cytotoxicity against the HCT-116 tumor cells [195]. Botryllamides exhibited selectivity toward BCRP, but have different specificity to other ABC transporters. Two naturally occurring botryllamides, botryllamide I (**89**) and J (**90**) were reported to have activity against BCRP by inhibiting BCRP-mediated BODIPY FL prazosin transport in BCRP-transfected HEK293 cells (Figure 18). Both **89** and **90** competed with [^125^I]-iodoaryl-azidoprazosin labeling of BCRP, and were shown to stimulate BCRP-associated ATPase activity, reversing BCRP-mediated resistance [196]. In a different study, botryllamide G (**91**) was reported as the most potent botryllamide BCRP inhibitor, but did not inhibit the efflux of rhodamine out of P-gp overexpressing cells. In contrast, botryllamide A (**92**), which is less potent against BCRP, showed that activity against P-gp [197]. These differences were explained by the present or absent of an *o*-methyl group at C-15. The biological investigation of analogs of botryllamides **92** and **93** suggested that the 2-methoxy-*p*-coumaric acid moiety and the number of conjugated double bonds with this group were important for BCRP inhibition [197]. This study also found that: (i) the phenolic hydroxyl group at C-7 was not important for activity; (ii) the binding region with BCRP was between C-4 and C-9 (right side of Figure 18) of the aryl ring; and (iii) conjugation through C-1 to C-9 was necessary for activity, but extended conjugation from C-10 to C-17 (left side of Figure 18) of the aryl ring was not required, however, it might contribute to specificity of activity.

**Scheme 17:**
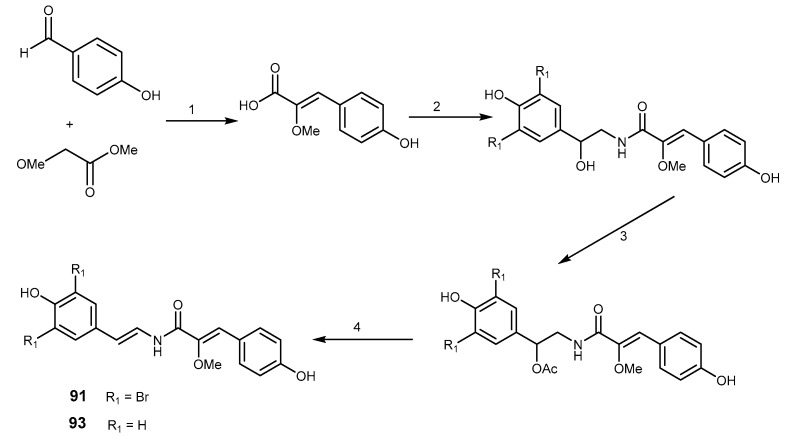
Synthesis of boryllamides G (**91**) and F (**93**). *Reagents and conditions*: (1) NaOMe, MeOH, reflux, overnight; (2) octopamine·HCl or bis-brominated octopamine, WSCl, HOBt, Et_3_N; (3) Ac_2_O, pyridine; (4) K_2_CO_3_, DMSO.

The syntheses of botryllamides G (**91**) and F (**93**) were already described, and are depicted in Scheme 17. The strategy started with a condensation of octopamine with 2-methoxy-*p*-coumaric acid to generate an amide bond, followed by a dehydration of the adjacent hydroxyl group to provide the key enamine amide functionality [197].

#### 2.6.3. Kendarimide

**Figure 19:**
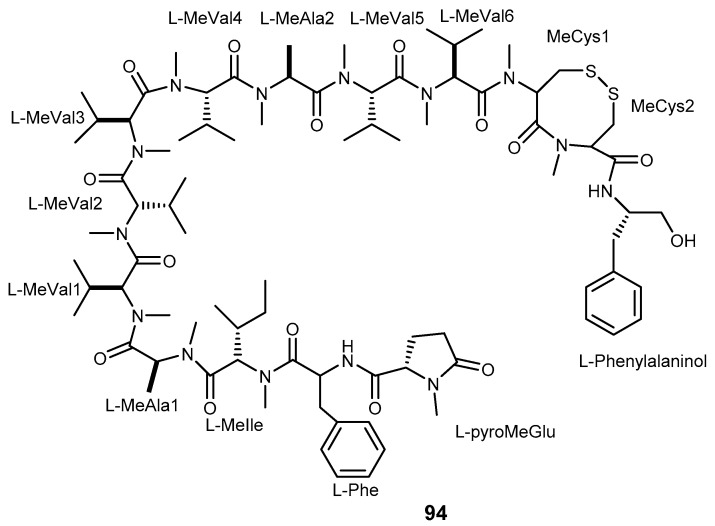
Structure of kendarimide A (**94**).

Kendarimide A (**94**, Figure 19) is an oligopeptide found in the Indonesian sponge *Haliclona* sp. This compound showed the potential of MDR reversal in human carcinoma (KB-C2) cell line overexpressing P-gp at the concentration of 6 μM, however, it was not active against KB-3-1 at the same concentration. The combination of **94** (6 μM) with colchicine (0.1 µg/mL) was found to inhibit KB-C2 cell growth by 87% [198]. Kendarimide A (**94**) is composed of several amino acids such as *N*-methylpyroglutamic acid (pyroMeGlu), *N*-methylated eight membered cysteinyl-cysteine (ox-[MeCys-MeCys]) together with many *N*-methyl amino acid residues, similar to cyclosporine A, a well-known P-gp inhibitor [199]. These data highlight that peptides can be considered as a valuable scaffold to reverse MDR.

**Scheme 18:**
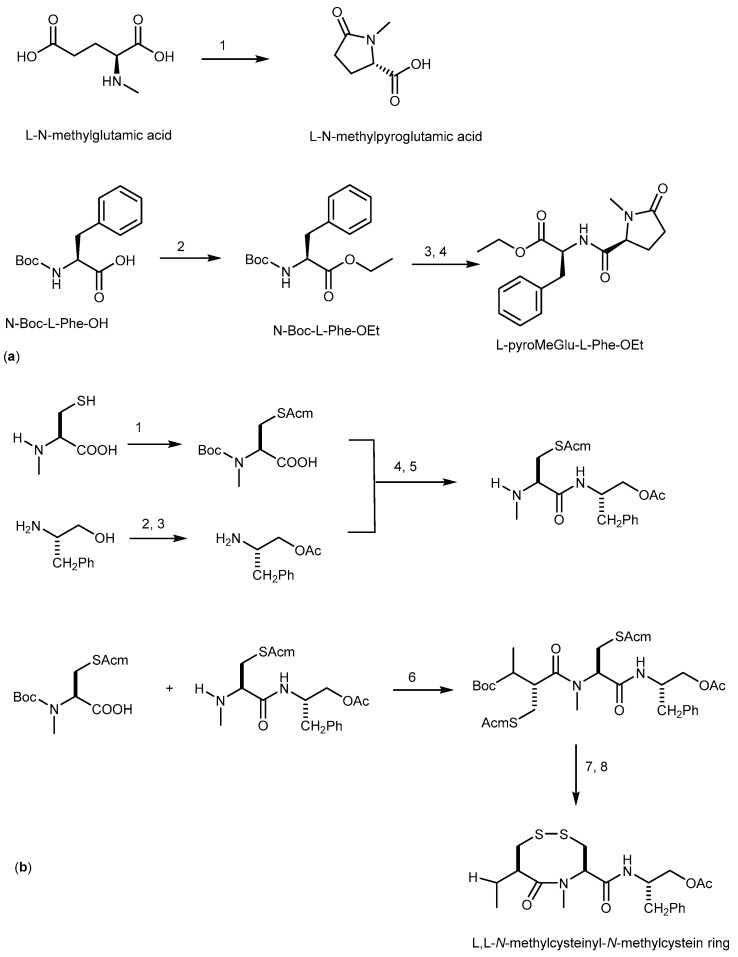
Synthesis of l-pyroMeGlu-l-Phe-OEt (**a**) and l,l-*N*-methylcysteinyl-*N*-methylcystein ring (**b**). Reagents and Conditions: (**a**) (1) H_2_O, 132 °C, 1.9 kg/cm^2^ (autoclave); (2) Na_2_HCO_3_, C_2_H_5_I, DMF; (3) TFA, CH_2_Cl_2_; (4) l-*N*-pyroMeGlu, DEPC, TEA, DMF, 0 °C; (**b**) (1) acetamidomethanol, conc. HCl, (Boc)_2_O, 1N NaOH; (2) (Boc)_2_O, 1N NaOH, Ac_2_O, pyridine; (3) TFA, CH_2_Cl_2_; (4) DEPC, Et_3_N, DMF; (5) TFA, CH_2_Cl_2_, quant.; (6) EDCI, HOAt, CH_2_Cl_2_/DMF; (7) I_2_, CH_2_Cl_2_/MeOH; (8) TFA, CH_2_Cl_2_, quant.

From our understanding the total synthesis of compound **94** has not yet been reported. l-pyroMeGlu-l-Phe-OEt is the moiety that was synthetized as a model compound to represent pyroMeGlu of **94** as described in Scheme 18a [198]. Another synthetic model compound (*N*-methylcysteinyl-*N*-methylcysteine ring) to study the absolute stereostructure of the C-terminal tetrapeptides (*N*-MeCys-*N*-MeCys) in **94** was synthesized, and the absolute configuration of both of the two adjacent *N*-methylcysteines in **94**, which form an eight-membered disulfide ring, was elucidated to be L. The synthetic pathway was described in Scheme 18b [199].

#### 2.6.4. Patellamide and Derivatives

**Figure 20:**
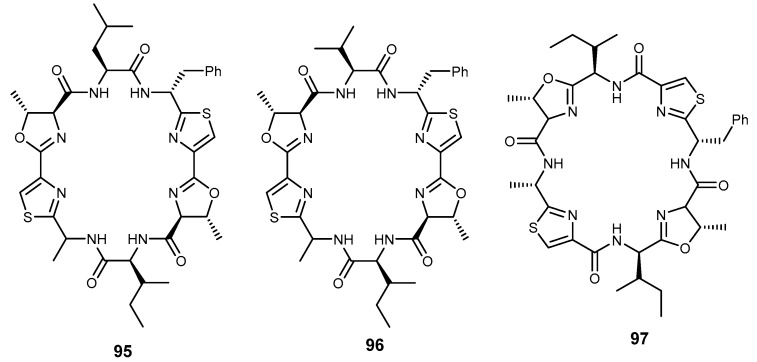
Structures of patellamides B (**95**), C (**96**), and D (**97**).

Patellamides are thiazole and oxazoline lipophilic cyclic peptides isolated from the tunicate *Lissoclinum patella*. These compounds exhibited cytotoxicity and reversed MDR in tumor cells. Patellamides B (**95**), C (**96**), and D (**97**) showed reversal activity and enhanced the activity of P-gp inhibitors such as vinblastine, colchicine, and doxorubicin in CEM/VLB100 cell line (Figure 20) [200,201]. 

**Scheme 19:**
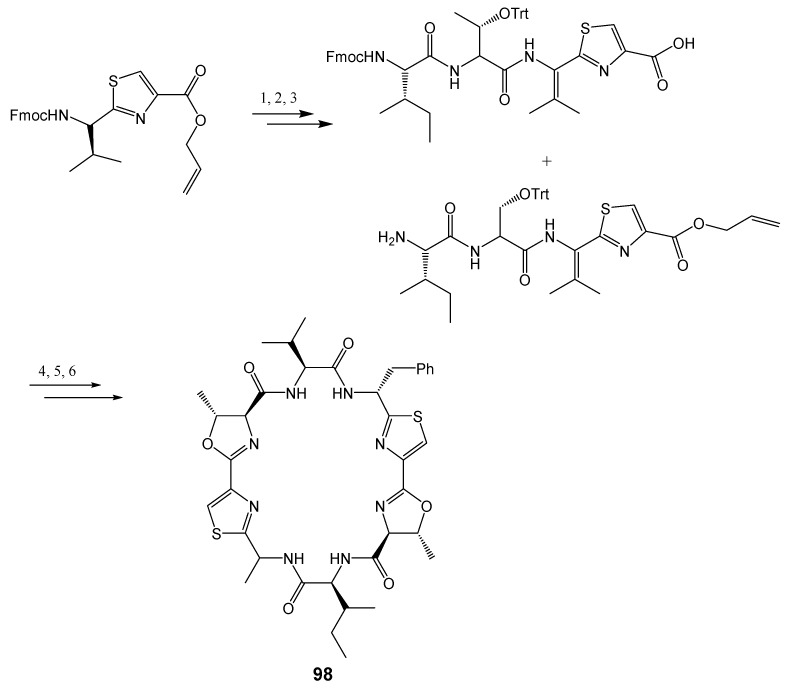
Synthesis of patellamide A (**98**). *Reagents and conditions*: (1) 20% piperidine/DMF, HOBt, HBTU, DIEA, Fmoc-aThr(Trt)-OH; (2) 20% piperidine/DMF, HOBt, HBTU, DIEA, Fmoc-Ile-OH; (3) Pd(PPh_3_)_4_, PhSiH_3_, CH_2_Cl_2_, 6 h and 20% piperidine/DMF; (4) HOBt, HBTU, DIEA, CH_2_Cl_2_, 2% TFA, PhSH, CH_2_Cl_2_; (5) Burgess reagent, THF, 55 °C, 1 h then 77 °C, 4 h; (6) Pd(PPh_3_)_4_, PhSiH_3_, CH_2_Cl_2_, 2 h.

Patellamide D (**97**) showed a better result than verapamil in modulating drug resistance in vitro, and colchicine cytotoxicity were enhanced by 2.8 fold. Doxorubicin toxicity was reduced from IC_50_ > 1000 ng/mL to 110 ng/mL, by **97**. This result indicated that patellamide D (**97**) acts as a selective antagonist in MDR, and thus can be considered as a potential modulator for drug resistance [7]. The synthesis of patellamide derivatives has been accomplished, using thiazole as starting material, via two contemporary heterocyclization approaches to form oxazolines and thiazoline via coupling and cyclodehydration reactions. The example illustrating the synthesis of patellamide A (**98**) is described in Scheme 19 [202]. 

### 2.7. Miscellaneous

#### 2.7.1. Irciniasulfonic Acid Derivatives

**Figure 21:**
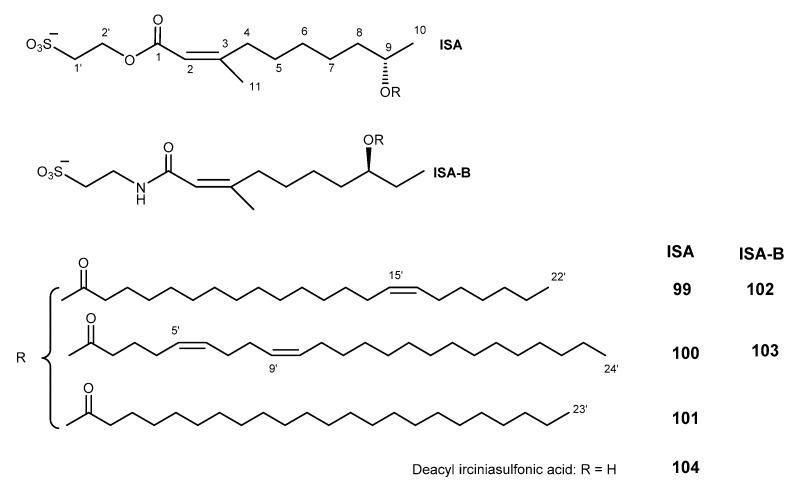
Structure of ISA (**99**–**101**), ISA-B (**102**, **103**), and deacyl ISA (**104**).

Irciniasulfonic acids consist of ISA (compounds **99**–**101**, [203]) and ISA-B (compounds **102**, **103**, [204]). These acids are esters that were isolated from the marine sponge *Ircinia* sp. [203], and deacyl irciniasulfonic acid was isolated from tropical *Coscinoderma* sp. sponge (Figure 21) [205]*.* Irciniasulfonic acids **99**–**103** were found to reverse MDR against KB/VJ300 overexpressing P-gp at the concentration of 100 μM in the present of vincristine [203,204,206]. Compound **99**–**101** were shown to reverse MDR against KB/VJ300 overexpressing P-pg in the presence of verapamil at the concentration of 25 μM, and the simplified analog (deacyl ISA, **104**) showed to be 13-fold more potent than other irciniasulfonic acids in reversal the MDR phenotype [203,204].

**Scheme 20:**
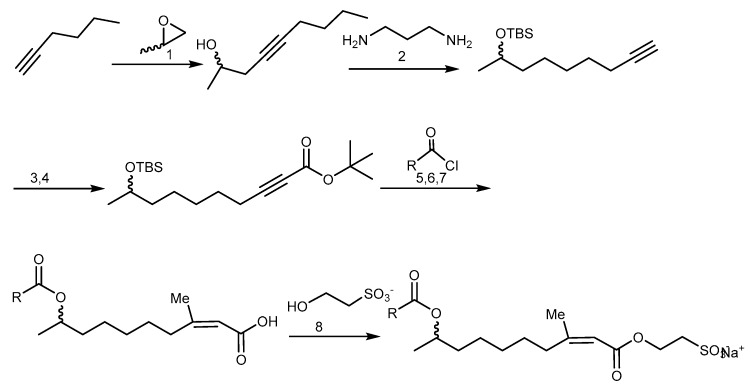
The total synthesis of ISA derivatives. *Reagents and conditions*: (1) *n*-BuLi, Et_2_AlCl, toluene; (2) KH; (3) TBS-Cl, imidazole, DMAP, DMF; (4) *n*-BuLi, Boc_2_O; (5) CuBr_2_.SMe_2_, MeLi, THF; (6) HF-pyridine; (7) TFA, CH_2_Cl_2_; (8) (COCl)_2_, DMF cat, CH_2_Cl_2_, toluene, reflux.

The synthesis of ISA derivatives was accomplished using propylene oxide as starting material via a convergent approach to achieve both enantiomers of the natural product and allowed also the incorporation of a range of side chains for optimization of their biological activity. The procedure of this synthesis is illustrated in Scheme 20 [207].

#### 2.7.2. Secalonic Acid D

**Scheme 21:**
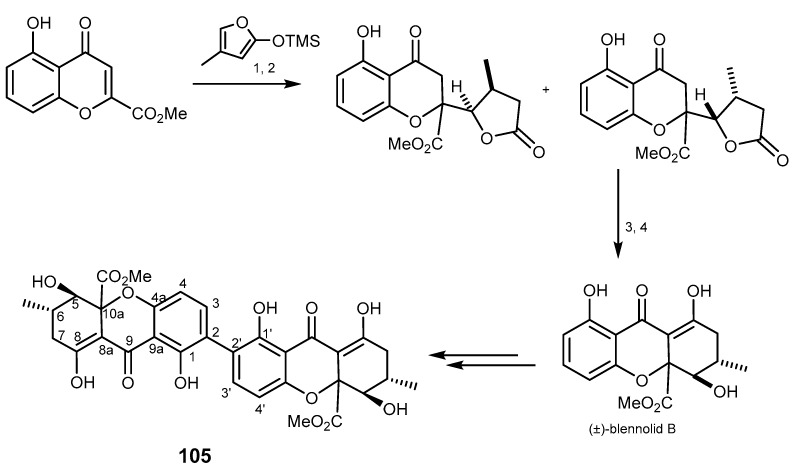
Total synthesis of secalonic acid D (**105**). *Reagents and conditions*: (1) 2,6-lutidine, *i*PrSi(OTf)_2_, CH_2_Cl_2_, then Et_3_N·3HF; (2) Rh/Al_2_O_3_, H_2_, MeOH; (3) NaH, THF; (4) CH_2_Cl_2_, then NaH, THF.

Secalonic acid D (**105**, Scheme 21) is a mycotoxin isolated from the marine fungi *Penicillium oxalicum* [208] and *Gliocladium* sp. T31 [209], which was investigated for antitumor properties as a DNA topoisomerase I inhibitor [210]. This compound showed a potent cytotoxicity on MDR cells (P-gp, MRP1, and BCRP-overexpressing cells) and on their parental cells by down-regulating the expression of BCRP protein [211]. It also decreased the percentage of side population cell in lung cancer cells. These results highlight **105** as an interesting molecule that has a potent cytotoxic activity by enduring BCRP degradation, and was considered as a lead compound for the development of new BCRP inhibitors [212].

Chemically, secalonic acid D (**105**) is the chiral dimeric natural product of ergochrome xanthone [213]. The synthesis of secalonic acid D (**105**) started from the intermediate compound (±)-blennolide B, which was synthesized from 5-hydroxychromone, with a construction of a hemisecalonic derivative, followed by the conversion with iodide and stannane. This reaction is a copper-catalyzed C–C bond-forming between the two molecules under oxidative conditions as the key dimerization step. This reaction is the first challenge to oxidize the xanthone framework which was previously shown as unsuitable for a direct oxidation. The iodide compound can be preactivated at an *o*-position to make the dimerization regioselective. The total synthesis of secalonic acid D (**105**) is illustrated in Scheme 21 [213,214,215,216].

#### 2.7.3. Terrein

**Scheme 22:**
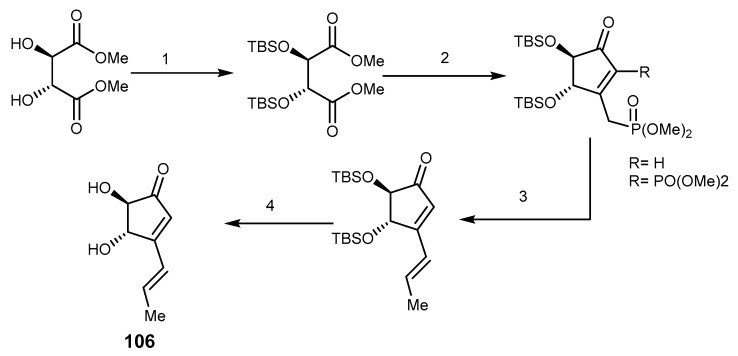
Synthesis of (+)-terrein (**106**). *Reagents and conditions*: (1) TBSCl, imidazole, DMF; (2) *n*-BuLi, MePO(OMe)_2_, THF, then benzene-H_2_O, reflux; (3) NaH, MeCHO, THF; (4) (Et_4_NCl), MeCN or HF·MeCN or TBAF, MeCN.

Terrein (**106**, Scheme 22) is a fungal metabolite isolated from the marine-derived thermophilic fungus *Aspergillus terreus.* This metabolite has been already reported to inhibit cell proliferation, to induce cell cycle arrest in human ovarian tumor cells (SKOV3,) and to inhibit the proliferation of ovarian cancer stem-like cells [217], to inhibit the epidermal proliferation of skin [218], as well as to inhibit melanogenesis [219]. It also displayed a strong cytotoxicity against breast cancer (MCF-7) cell and suppressed the growth of MCF-7 expressing BCRP cells by inducing apoptosis caspase-7 pathway, and inhibiting the Akt signaling pathway [220].

A simple synthesis of (+)-terrein (**106**) was reported by using l-tartrate or dimethyl l-tartrate as starting materials. The whole process included cyclization, Horner-Wadsworth-Emmons, and deprotection of bis-*t*-butyldimethylsilyl (bis-TBS) group as shown in Scheme 22 [221].

#### 2.7.4. Shornephine A

**Figure 22:**
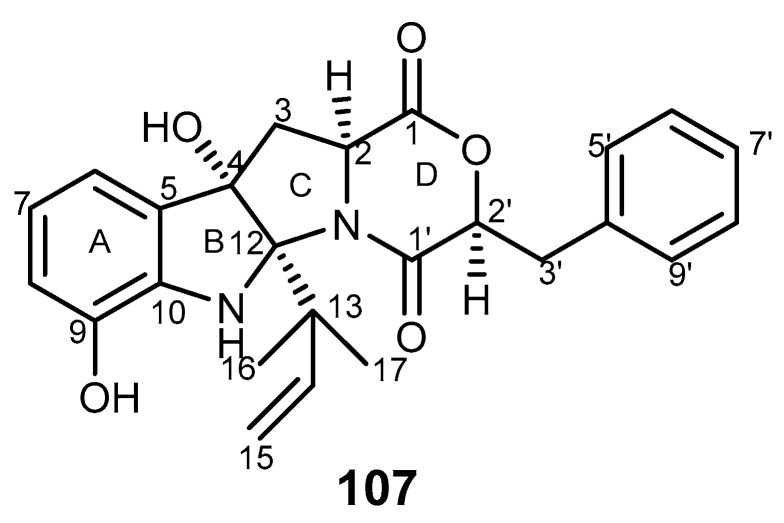
Structure of shornephine A (**107**).

Shornephine A (**107**, Figure 22) is a diketomorpholine isolated from the marine fungus *Aspergillus* sp. (CMC-M081F) which was collected from marine sediment. This compound was described as non-cytotoxic against bacteria, fungus, and tumor cell lines and an inhibitor of P-gp in MDR human colon tumor cells (SW60 Ad300) at the concentration of 20 μM [222].

## 3. Future Perspectives

We are now witnessing a renaissance of the interest in efflux transporters, not only due to regulatory requirements but also to the significant role of these carriers in the adsorption, distribution, metabolism, excretion, and toxicity (ADMET) process of drug discovery. Therefore, P-gp and other ABC transporters that mediate drug efflux are recognized as a “team to beat”. Marine organisms have proven to be an important source in the discovery and development of interesting compounds, and, in particular, of anticancer agents. These marine-derived compounds, which can be grouped mainly as alkaloids, polyoxygenated sterols, terpenoids, and peptides, have demonstrated a reversal effect of MDR by themselves. Some compounds were shown to increase rather than decrease cytotoxicity towards MDR cell lines and can be defined as collateral sensitizing agents or this effect was shown in combination with anticancer drugs, being non-cytotoxic MDR reversal agents. The major sources of marine MDR reversal agents and mechanisms have been scarcely studied, and very little is known about their mechanism of action. Several marine compounds which have shown potent and selective MDR activity against cancer cell lines belong to the alkaloids; however, they present some toxicity. Among marine natural products, trabectedin has proved to be the most promising against MDR with in vitro and in vivo efficacy. Chemically, synthetic analogs of marine compounds acting as inhibitors have been investigated to achieve higher activity, less toxicity and less pharmacokinetic interaction properties, and to establish SAR. With this diversity of scaffolds it is not possible to extract common features essential for MDR reversal activity; however, most of the derivatives are highly lipophilic and contain fused rings and multiple chiral centers, which in turn reflect their complex total synthesis procedures. Among the synthetic analogs, eriburin has been shown to be the most promising P-gp modulator, active against MDR in vitro and with proved safety in clinical patients. These features also open new challenges for medicinal chemists to accomplish viable synthetic routes and new avenues in the design of analogs with suitable drug-like properties that could render potential drug candidates. Joining together all the pieces of the chemical-biological-pharmaceutical puzzle, we can anticipate more effective chemotherapies based on molecules from the sea.
